# High efficiency blue organic light-emitting diodes with below-bandgap electroluminescence

**DOI:** 10.1038/s41467-021-25135-z

**Published:** 2021-08-11

**Authors:** Maria Vasilopoulou, Abd. Rashid bin Mohd Yusoff, Matyas Daboczi, Julio Conforto, Anderson Emanuel Ximim Gavim, Wilson Jose da Silva, Andreia Gerniski Macedo, Anastasia Soultati, George Pistolis, Fabio Kurt Schneider, Yifan Dong, Polina Jacoutot, Georgios Rotas, Jin Jang, Georgios C. Vougioukalakis, Christos L. Chochos, Ji-Seon Kim, Nicola Gasparini

**Affiliations:** 1 Institute of Nanoscience and Nanotechnology, National Centre for Scientific Research Demokritos, Terma Patriarchou Grigoriou, Agia Paraskevi, Greece; 2 Department of Chemical Engineering, Pohang University of Science and Technology (POSTECH), Pohang, Gyeongbuk Republic of Korea; 3 Department of Physics and Centre for Processable Electronics, Imperial College London, London, UK; 4 Universidade Tecnologica Federal do Parana, GPGEI, Curitiba, Parana Brazil; 5 Department of Chemistry and Centre for Processable Electronics, Imperial College London, London, UK; 6 Department of Chemistry, National and Kapodistrian University of Athens, Athens, Greece; 7 Advanced Display Research Center, Department of Information Display, Kyung Hee University, Dongdaemoon-gu, Seoul, South Korea; 8 Institute of Chemical Biology, National Hellenic Research Foundation, Athens, Greece

**Keywords:** Electronic devices, Organic LEDs

## Abstract

Blue organic light-emitting diodes require high triplet interlayer materials, which induce large energetic barriers at the interfaces resulting in high device voltages and reduced efficiencies. Here, we alleviate this issue by designing a low triplet energy hole transporting interlayer with high mobility, combined with an interface exciplex that confines excitons at the emissive layer/electron transporting material interface. As a result, blue thermally activated delay fluorescent organic light-emitting diodes with a below-bandgap turn-on voltage of 2.5 V and an external quantum efficiency (EQE) of 41.2% were successfully fabricated. These devices also showed suppressed efficiency roll-off maintaining an EQE of 34.8% at 1000 cd m^−2^. Our approach paves the way for further progress through exploring alternative device engineering approaches instead of only focusing on the demanding synthesis of organic compounds with complex structures.

## Abstract (web summary)

Thermally activated delayed fluorescence organic light-emitting diodes (TADF-OLEDs) rely on high triplet energy interlayers to confine excitons, which results in reduced performance. Here, the authors report high-performance blue TADF-OLEDs with below bandgap electroluminescence.

## Introduction

Organic light-emitting diodes (OLEDs) with blue emission of high efficiency and operational stability are very important in solid-state lighting, information storage of high density, and vivid displays^1–5^. The low-efficiency issue of purely fluorescent devices was successfully addressed by the development of noble metal-based organometallic phosphors that enabled the fabrication of blue OLEDs with external quantum efficiencies (EQEs) exceeding 30%^6–9^. However, the high cost and limited reserve of noble metal phosphors have been the driving force for developing purely organic thermally activated delay fluorescent (TADF) emitters relying on radiative deactivation from singlet states with 100% quantum efficiency^10–13^. Through careful design and compositional engineering of such emitters^14–17^, the EQEs of blue and sky-blue TADF OLEDs have been steadily improved from 19.5% in 2014^18^ to 38.2% in 2019^19^ and further to 38.4% in 2021^20^, hence surpassing in performance the best blue phosphorescence OLEDs^9^. These devices to successfully confine both charges and excitons within the blue emitter, which is generally dispersed into an appropriate matrix to form the emissive layer (EML), require high lowest unoccupied molecular orbital (LUMO) and triplet energy (E_T_) interlayers^21^. As a result, large energetic barriers are present at the interfaces resulting in high device voltages, reduced efficiencies, and high efficiency roll-off due to the carrier accumulation at these interfaces.

In a standard OLED architecture, the electrical carrier injection takes place via the highest occupied molecular orbital (HOMO) and the LUMO of the hole injection layer (HIL) and electron injection interlayer (EIL) injection interlayer, respectively. To reduce the charge injection barriers, it is necessary to adjust the energy levels of the HIL/EIL with the work function (W_F_) of the respective electrode. However, to confine excitons (both singlet and triplet) within the blue EML, interlayer materials with high singlet energy and E_T_ (commonly above 3.0 eV^22^) are necessary. These requirements are met in blue OLEDs by fabricating multi-layer stacks, including separate charge injection, transporting, and exciton-confining interlayers. These stacks include several interfaces where charges and excitons are accumulated, deteriorating the device performance and causing a drop of EQE in high current densities (*J*) known as efficiency roll-off^23–25^. One way to prevent these parasitic effects is, therefore, the design and fabrication of simplified structures^26–28^.

Here we apply a device-engineering approach that allowed us to demonstrate blue TADF OLEDs with high figure of merits, including below bandgap turn-on voltage of 2.5 V and the highest reported to date EQE for any blue OLED of 41.2%. The high OLED performance was made possible through the application of a low E_T_ (2.14 eV) hole transport material (HTM), rationally designed with a high hole mobility of 7.5 × 10^−3^ cm^2^ (V s)^−1^. This HTM served as a hole “reservoir” influencing both hole and electron carrier injection, and transport and subsequent exciton formation at the EML/electron transport material (ETM) interface. Effective exciton confinement due to the formation of an interface exciplex between the ambipolar host and the ETM prevents exciton diffusion towards the low E_T_ HTM, resulting in minimal energy loss, below-bandgap electroluminescence (EL), high efficiency, improved lifetime, and reduced efficiency roll-off.

## Results

### Materials and device conceptual design

**Fig. 1:**
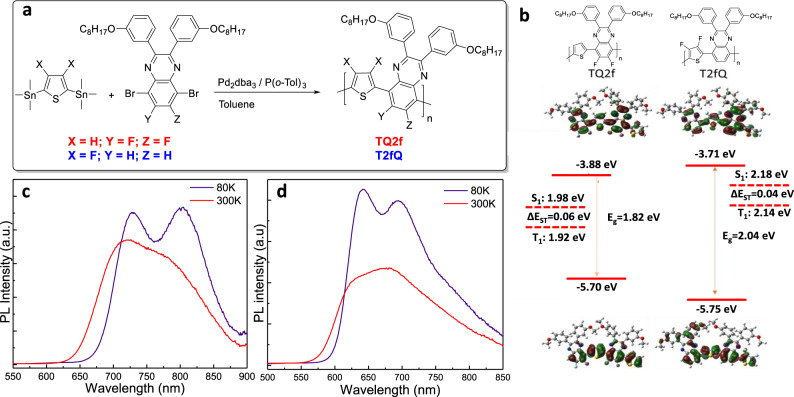
Properties of the hole-transporting interlayers. **a** The synthetic procedure for the preparation of fluorinated TQs. **b** The chemical structures of TQ2f and T2fQ used as hole-transporting interlayers, the calculated frontier orbitals and energy levels as derived from cyclic voltammetry, and absorption measurements and the lower excited singlet and triplet levels of TQ2f and T2fQ. The room temperature (RT, 300 K) and low temperature (80 K) photoluminescence spectra of **c** TQ2f and **d** T2fQ thin films.

Besides technical specifications, any developed material for OLED applications needs to be able to scale up to hundreds of kilograms, at an acceptable cost and environmental impact for real-world applications. Thiophene-quinoxaline (TQ) copolymers are considered very promising organic photovoltaic donor materials of low synthetic complexity and easy scale-up at multigram quantities^29^. In this work, we synthesized two fluorinated-TQ copolymers, abbreviated as T2fQ and TQ2f, containing two fluorine (F) atoms in thiophene and quinoxaline, respectively. The synthetic procedure is shown in Fig. 1a. The chemical structure of each copolymer is shown in the upper panel of Fig. 1b. The identity, purity, molecular weight, and optoelectronic properties of both compounds were ascertained using ^1^H and ^13^C nuclear magnetic resonance (^1^H-NMR and ^13^C-NMR, respectively), gel permeation chromatography (GPC), absorption and emission spectroscopy, and cyclic voltammetry during oxidation (Supplementary Figs. 1–4 and Supplementary Tables 1 and 2). The latter enabled the estimation of the fluorinated TQ’s HOMO, whereas absorption measurements defined the optical bandgap value of each copolymer. The calculated frontier orbitals and the corresponding energy levels (Supplementary Note 1) are presented in Fig. 1b. The replacement of the F atoms from the quinoxaline to the thiophene has significant effect on the energy levels along with the dihedral angle between the two adjustments building blocks (Supplementary Fig. 5). This is expected to largely affect the intramolecular charge transfer and molecular packing, and, consequently, the carrier mobility of these copolymers in the solid state^30–32^. Photoluminescence (PL) spectra and transient PL (TRPL) decay curves measured either in ambient at a wide temperature range from 80 K to room temperature (300 K, RT) (Fig. 1c, d and Supplementary Figs. 6 and 7) or in oxygen/nitrogen environments (at RT) (Supplementary Figs. 8 and 9) enabled the estimation of the lowest excited singlet (S_1_) and triplet (T_1_) states (Fig. 1b), while also indicating that these HTMs are RT phosphorescence materials. From the energy levels point of view, these materials are far from being ideal interlayers for direct contact with the EML, where requirements for high LUMO and high E_T_ to avoid electron leakage and prevent exciton quenching are very strict.

Contrarily to conventional OLED architectures where excitons are directly formed in the emitter, OLEDs using donor : acceptor (D : A) species interacting in the excited state, the so-called exciplexes, have gained much attention after the pioneering work of Adachi and colleagues^17,33^. Analogously to TADF emitters, exciplexes display an innately small energy separation (ΔΕ_ST_) between their charge-transfer excited states (these are either singlet, ^1^CT, or triplet, ^3^CT, in character), indicating a feasible way toward theoretical 100% exciton harvesting^17^, when applied either as hosts or as emitters^33^. As an alternative strategy, the use of interface exciplexes to strongly localize excitons and improve OLED efficiency has also been reported^22^, albeit there are stringent requirements that should be fulfilled for optimum device performance, especially for blue OLEDs^34^. In particular, ^1^CT/^3^CT states have to be lower than the local excitation energies (^1^LE/^3^LE) of both donor and acceptor constituents for the successful confinement of all excitons within the exciplex. At the same time, ^1^CT/^3^CT states must be higher than the emitter’s LE states in order to succeed efficient energy transfer towards the latter at RT and avoid back-electron transfer towards the donor or acceptor constituents. In blue OLEDs, in particular interface exciplexes with high ^1^CT/^3^CT states lying above those of the blue emitters (~3.0 eV^9^) are required, which necessitates the selection of large bandgap donor and acceptor constituents. We rationally selected an ambipolar host, namely bis(diphenylphosphine oxide)dibenzofuran (DBFPO)^35^ (Supplementary Note 2 and Supplementary Figs. 10–12), which served as the donor constituent combined with an ETM, namely diphenyl[4-(triphenylsilyl)phenyl]phosphine oxide (TSPO1)^36^, to serve as the acceptor constituent of the interface exciplex. DBFPO bears diplenylphosphine oxide acceptor and dibenzofuran donor groups; TSPO1 consists of triplenyl silyl and triphenyl oxide acceptor groups. The multiple *π*–*σ* interactions through C-Si direct coupling substantially lower its HOMO energy, rendering this compound an excellent conventional acceptor^37^.

**Fig. 2:**
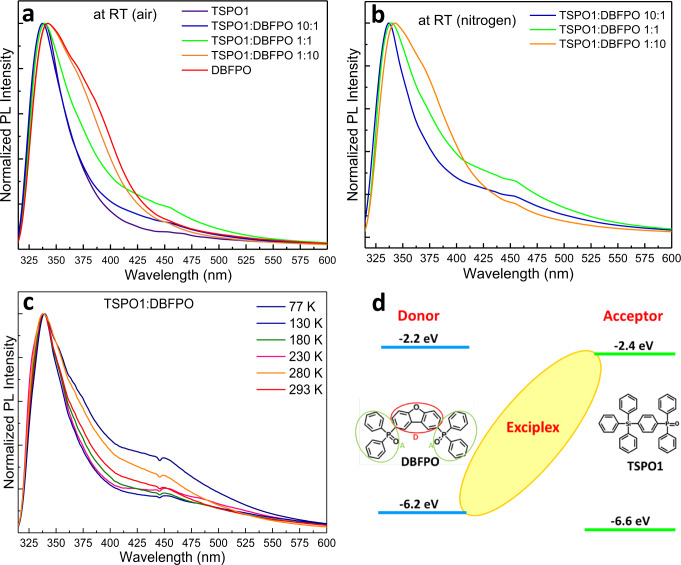
Illustration of the interface exciplex formation. **a** Room temperature (RT) normalized PL spectra of TSPO1 and DBFPO pristine films and mixtures. **b** The PL spectra of mixtures taken at RT in nitrogen. **c** Temperature-dependent PL spectra of TSPO1:DBFPO 1:1 mixed film. **d** The chemical structures and frontier orbitals of DBFPO and TSPO1, and the illustration of interfacial exciplex formation.

We investigated the possible exciplex formation between TSPO1 and DBFPO constituents by monitoring the absorption and emission spectra of the monomers in solution and of pristine and mixed films. Supplementary Fig. 13 presents the absorption and emission spectra of TSPO1 and DBFPO monomers in dilute solution (5 × 10^−7^ M) and films. The PL spectrum of DBFPO film exhibits a red-shifted broad shoulder, which is attributed to excimer emission (in accordance to ref. ^35^). By subtracting the PL spectrum of the monomer, we found that this emission is centered at 361 nm, which is in well agreement with previous reports^35^. We next investigated the absorption and emission properties of mixed (in molecular ratio of 10:1, 1:1, and 1:10) TSPO1 and DBFPO films. As depicted in Supplementary Fig. 14, their absorption spectra derive from the superposition of the constituents, which indicates that these materials do not interact in the ground state. Differently, the PL spectra of the mixed films exhibit strict differences; we note that these spectra were taken using a 330 nm short-pass filter to cut most of the ultraviolet (UV) emission of the pristine materials and focus on the longer wavelengths where exciplex formation—if any—is expected. In the spectra taken in ambient conditions at RT (Fig. 2a), in addition to the contribution of the monomers and DBFPO’s excimer emission, which appears as a shoulder at ~361 nm in the 1:1 ratio and mostly in the 1:10 film with the higher DBFPO content, a new distinct peak appears at longer wavelengths (centered at 440–450 nm). This peak exhibits higher intensity in the spectrum of 1:1 mixed film, where larger interaction between the two constituents is expected. Notably, this peak becomes even more pronounced in the spectra taken in nitrogen (Fig. 2b) and at low temperatures (Fig. 2c). The Gaussian peak deconvolution (Supplementary Fig. 15) revealed that the PL spectrum of the 1:1 mixed film can be considered as the superposition of narrow peaks derived from the monomers, one broad peak centered at 370 nm, which is related to the DBFPO’s excimer, and one broad peak centered at 440 nm. The latter is due to excited state interaction between TSPO1 and DBFPO, and can be attributed to exciplex formation between the constituents. This is in agreement with the higher intensity of this peak observed in the 1:1 film compared to the 1:10 and 10:1 films in air, as well as the increased intensity of the peak in nitrogen and at low temperature in the 1:1 mixture. The broad peak at ~440 nm is therefore attributed to charge-transfer emission from the complex excited dimer.

The PL studies of pristine compounds and mixed films revealed the exciplex formation between the two constituents. The higher energy peak in the PL spectrum of the mixed films is the remaining emission of the constituents due to incomplete energy transfer between them due to low energy difference (ΔΕ) between their LUMOs (0.2 eV, Fig. 2d). A Δ*Ε* > 0.5 eV is needed to allow 100% charge transfer between the consituents^38^. However, even in this case the formation of an interface exciplex might be beneficial for the OLED operation, as it is expected to confine excitons within the EML/ETM interface. These excitons can then be transferred to the emitter’s molecules instead of being diffused towards the HTM/EML interface where excitons are probably quenched. Our studies excluded the possibility that the PL broadening in the 1:1 mixed film is due to different orientation/conformation of the constituent molecules in the mixed film. Supplementary Fig. 16 and Fig. 2c present the evolution of PL spectra of TSPO1 and DBFPO pristine and their mixtures films upon increasing temperature. In pristine TSPO1 and TSPO1:DBFPO 10:1 films, the phosphorescence of the ETM is clearly observed at low temperature in the wavelength range of interest (around 400–550 nm). Upon heating to reach RT, TSPO1 exhibits no distinct emission, whereas the 10:1 film presents a small but distinct shoulder at 440 nm. Differently, the DBFPO and 1:10 films present a broad red-shifted shoulder (reaching up to 600 nm) in both films, especially in pristine DBFPO. This could be due to molecular rearrangement upon heating or the induction of photochemical processes from continuous excitation. A similar behavior was previously observed for DBFPO upon UV exposure in air^35^, which was also verified by our experiments (Supplementary Fig. 17a). This can be attributed to significant molecular rearrangement of this material upon heating/exposure and/or due to photochemical processes^35^. Contrarily to pristine DBFPO, negligible change was observed in the TSPO1:DBFPO 1:1 upon UV irradiation (Supplementary Fig. 17b), which represents a clear indication of strong molecular interactions between the excited states of the constituents in the mixed film. This supports the exciplex formation between different molecules rather than the interactions/rearrangements of similar molecules.

### Peformances of blue TADF OLEDs

**Fig. 3:**
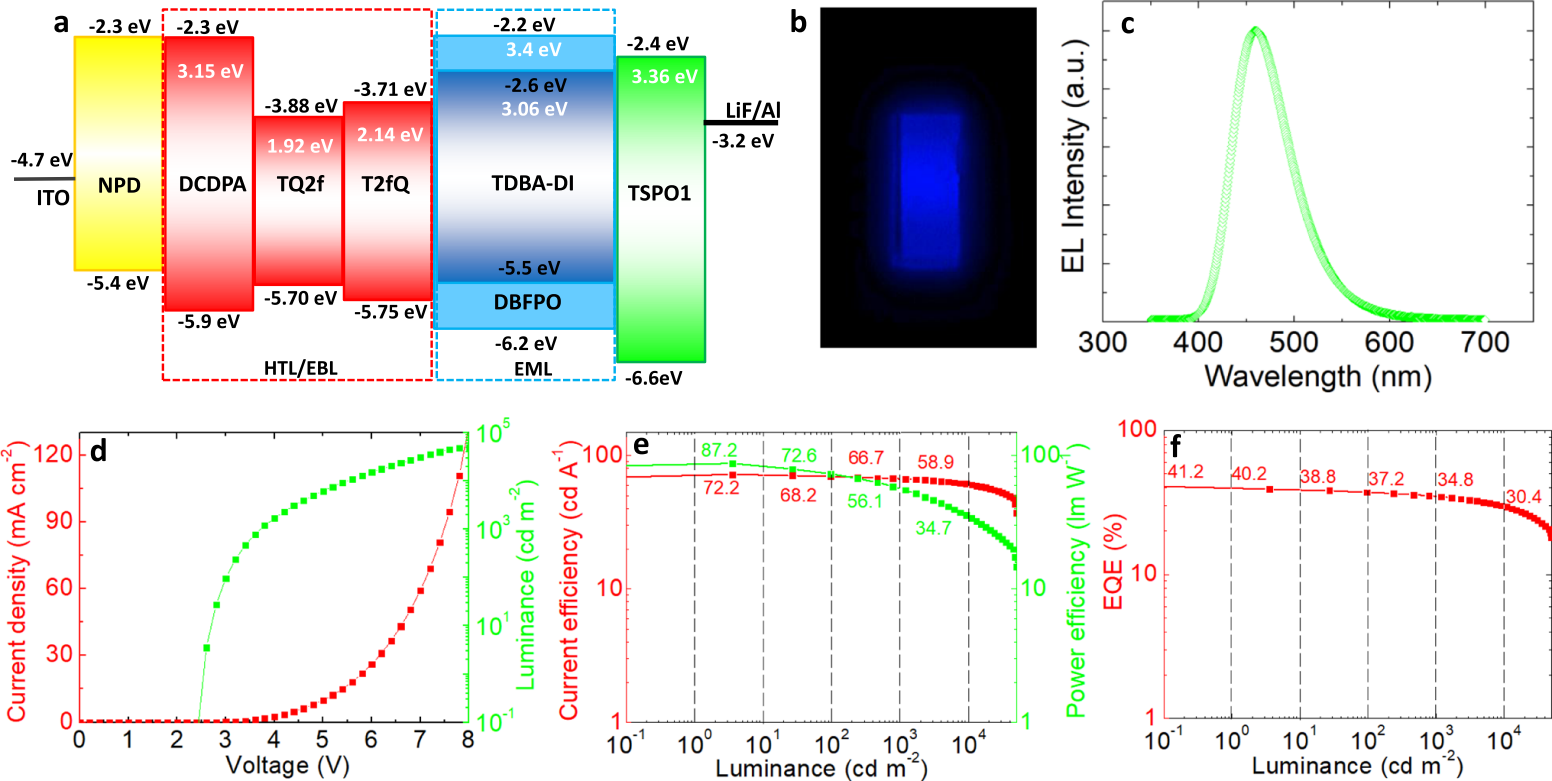
Performance of optimized blue TADF OLEDs and efficiency roll-off. **a** The energy-level diagram of the layers sequence in the blue TADF OLED device architecture considering vacuum level alignment before contact. Besides HOMO and LUMO energies of organic layers, the triplet energy of the emitter, host, and interlayer materials are also presented. **b** Photograph of a working OLED pixel with an active area of 25 mm^2^ and **c** electroluminescence spectrum of the same device. **d** Current density–luminance–voltage characteristics, **e** current and power efficiency vs. luminance, and **f** EQE vs. luminance characteristics for the OLED.

To test our conceptual device-engineering approach, we next fabricated blue OLEDs based on the 5-(2,12-di-tert-butyl-5,9-dioxa-13b-boranaphtho[3,2,1-de]anthracen-7-yl)-10,15-diphenyl-10,15-dihydro-5H-diindolo[3,2-a:3′,2′-c]carbazole (TDBA-DI) TADF emitter that previously enabled the fabrication of blue OLEDs with an EQE of 38.2%^19^. Our devices consisted of the following: indium tin oxide (ITO) anode, N,N′-Di(1-naphthyl)-N,N′-diphenyl-(1,1′-biphenyl)-4, 4′-diamine (α-NPD) hole injection layer (HIL), fluorinated TQ or 3,5-di(9H-carbazol-9-yl)-N,N-diphenylaniline (DCDPA) HTM^19^, 20% TDBA-DI doped in DBFPO host, TSPO1 ETM, and LiF/Al cathode. All device layers were vacuum evaporated, except for TQ derivatives, which were processed from tetrahydrofuran (THF) solution in order not to dissolve the NPD underneath. The solution concentration and processing conditions were optimized to obtain smooth films and maximum device performance (Supplementary Fig. 18). Figure 3a presents the frontier orbitals of organic materials and W_F_ values of metal contacts, considering energy vacuum level alignment before contact. It indicates that TQs are inappropriate for use as HTM in direct contact with the EML, as they could facilitate electron leakage and exciton quenching at the HTM/EML interface. Despite the negative prevision, the T2fQ device exhibited blue emission with color coordinates of (0.15, 0.28) (Fig. 3b), peak EL at 458 nm (Fig. 3c), and ultra-low turn-on voltage of 2.5 V (Fig. 3d), a current efficiency of 72.2 cd A^−1^, and a power efficiency (PE) of 87.2 lm W^−1^ (Fig. 3e and Table 1). The EL spectrum was identical with the PL one (Supplementary Fig. 19a), which suggests that T2fQ and DBFPO:TSPO1 interface exciplex have no contribution to the emitted wavelengths. The low device turn-on voltage corresponds to below-bandgap EL. The origin of such emission has been previously attributed to CT excitons present at the EML/ETM interface under sub-bandgap driving conditions^39^. The interface exciplex having CT states lying well below the emitter’s LE states (the 440 nm peak emission of the interface exciplex corresponds to CT energy of 2.8 nm, which is below the emitter’s LUMO, Fig. 3a), could contribute to the below-bandgap EL and explain the low turn-on voltage. Moreover, our device achieved a world record EQE of 41.2% (Fig. 3f), with a mean EQE of 34.6% (Supplementary Fig. 19b). Remarkably, a very low-efficiency roll-off of 8.6% at 1000 cd m^−2^ and 21.6% at 5000 cd m^−2^ was also obtained. Notably, the performance of the reference devices using the high-energy LUMO/high E_T_ DCDPA was slightly inferior to that of the T2fQ OLEDs (Supplementary Fig. 20). These devices achieved an EQE of 37.6%, which is very close to the efficiency of 38.2% exhibited by the previously reported OLEDs using DCDBA/TDBA-DI:DBFPO interfaces (Table 1)^19^. Notably, they still exhibited a below-bandgap turn-on voltage of 2.5 V (compared to 3.1 V of ref. ^19^), resulting in a higher PE of 82.1 lm W^−1^ (compared to 57.2 lm W^−1^ of ref. ^19^). However, the device using TQ2f was far inferior with an EQE hardly reaching 11.75% (Supplementary Fig. 21), an indication that the mobility of the HTM plays a crucial role in the device performance.

**Table 1:** Summary of the performance characteristics of our best-performing blue OLEDs and comparison with the best-performing, to date, blue TADF OLEDs^19,20^ and blue PHOLED^9^.

HTM//ETM	EML/*λ*_em_ (nm)	Turn-on voltage (V)^a^	FWHM (nm)^b^	Maximum luminance (cd m^−2^)	Maximum efficiency			CIE 1931 (*x*, *y*)^c^	Ref.
					CE (cd A^−1^)	PE (lm W^−1^)	EQE (%)		
DCDPA//TSPO1	DBFPO : TDBA-DI/458	2.5	65	37590	68.0 (48.0)	82.1 (26.3)	37.8 (29.0)	(0.15, 0.28)	This work
T2fQ//TSPO1	DBFPO : TDBA-DI/458	2.5	65	49530	72.2 (61.7)	87.2 (36.8)	41.2 (34.6)	(0.15, 0.28)	This work
DCDPA// DBFPO/TPBi	DBFPO : TDBA-DI/458	3.1	65	47680	64.4 (57.0)	57.2 (33.0)	38.2 (34.3)	(0.15, 0.28)	Ref. ^19^
mCP//DPEPO	DspiroS-TRZ/480	2.9	70	18446	90	101	38.4	(0.176, 0.374)	Ref. ^20^
^d^mCP//TmPyPB	CbBPCb : FIrpic/473	−	75	7000	53.6	50.6	30	−	Ref. ^9^

*CE* current efficiency, *PE* power efficiency.

^a^Turn-on voltage at 1 cd m^−2^.

^b^FWHM at 10 mA cm^−2^.

^c^CIE 1931 coordinates.

^d^Best phosphorescence OLED.

**Fig. 4:**
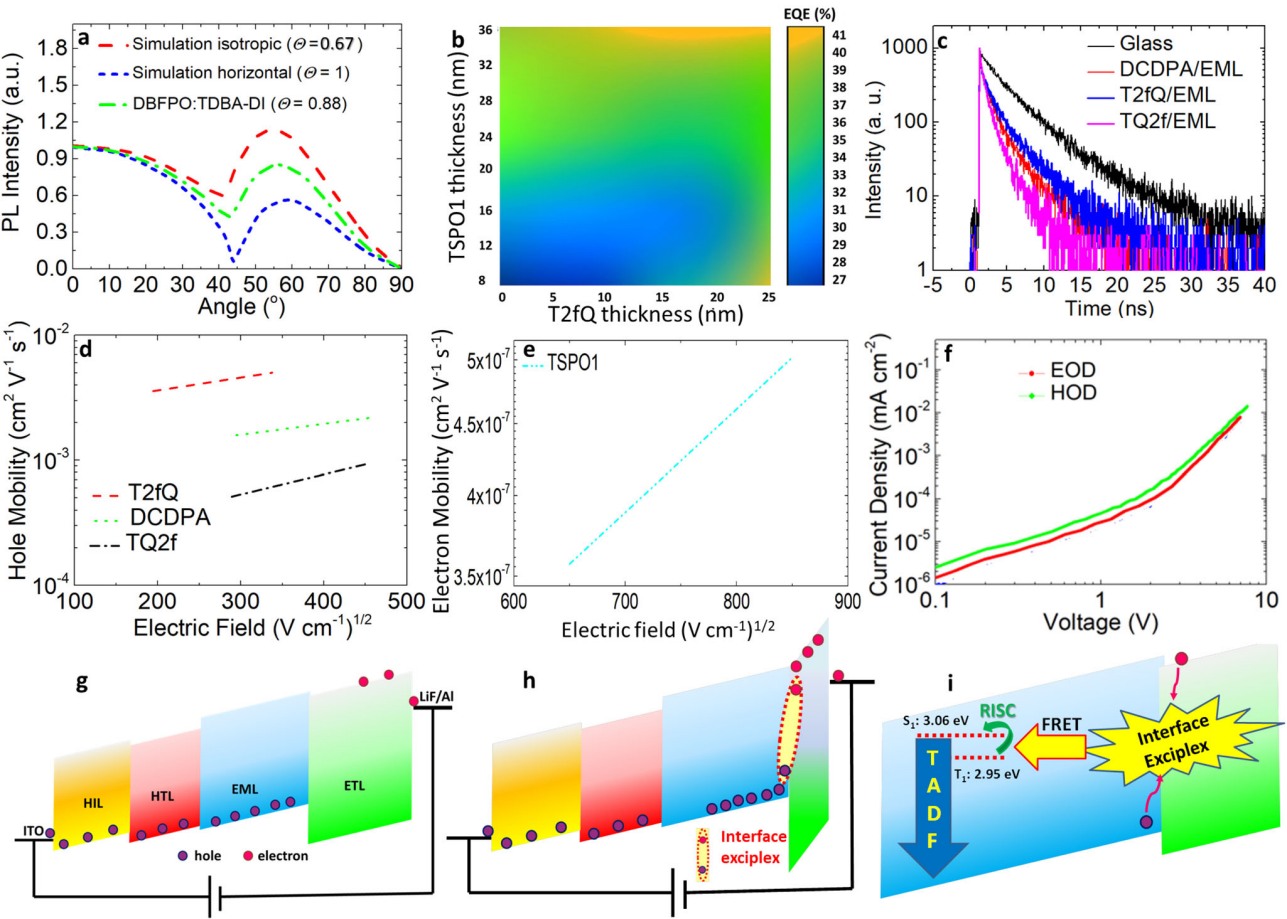
Proposed operation mechanism. **a** Dependence of polarized PL intensity on the emission angle and **b** simulations for the thickness optimization of TSPO1 and T2fQ ETM and HTM. **c** TRPL decay curves of the EML on glass, DCPPA, T2fQ, and TQ2f films. **d** Variation of hole mobility vs. the applied field for the three different HTMs used in this study. **e** Variation of electron mobility on the applied field of the TSPO1 ETM. **f** Current densities measured in hole-only (ITO/NPD/TQ2f/DBFPO 20%-TDBA-DI/NPD/Al) and electron-only (ITO/TSPO1/DBFPO 20%-TDBA-DI/TSPO1/LiF/Al) devices. Schematic response of an ITO/NPD/T2fQ/EML/ETL/LiF/Al device under forward applied bias. **g** The electric field (slope of the energy levels) is evenly distributed across the device. Holes and electrons are injected from the anode and cathode contact, respectively, and transported through the HTL and ETL towards the EML. Due to the large difference in hole and electron mobilities of the HTM and ETM, respectively, holes are reaching the EML before electrons are transported within the ETL. **h**, **i** Holes reaching and start building up at the EML/ETL interface screen the electric field to the ETL. The redistribution of the electric field into the ETL accelerates electron injection/transport and electrons reach the EML/ETL interface where they subsequently form interfacial exciplexes with holes in the DBFPO layer. Interface exciplex confines excitons and transfers them to the emitter.

To shed light to OLED performance, we next tried to determine the emitting dipole orientation within the EML of the champion OLEDs by performing angle-resolved PL measurements in a 30 nm film consisting of TDBA-DI 20%-doped in DBFPO. Figure 4a includes polarized PL measurements and optical simulations for isotropic oriented (Θ_//_ = 0.67) and horizontally oriented (Θ_//_ = 1) emitters. In general, horizontal emitting dipole (Θ_//_ = 1) show stronger light emission at the front direction, hence improving outcoupling efficiency^40,41^. The results shown in Fig. 4a confirm that when the EML is deposited on T2fQ, the larger portion of the emitting dipoles is horizontally oriented (Θ_//_ = 0.88). This is in good agreement with ref. ^19^, indicating that T2fQ underlayer preserves the same horizontal orientation of the emitting dipoles in EML as the previously applied DCDPA does. This result, however, cannot explain the best performance of the T2fQ-based devices compared to DCDPA ones, even though careful adjustment of HTM and ETM thicknesses enabled the achievement of the highest possible EQE (Fig. 4b). Aiming to reveal the origin of such remarkable device performance, we also measured the PL quantum yields in 20% doped TDBA-DI:DBFPO films deposited on DCDPA, TQ2f, and T2fQ layers; the corresponding values were quite close, in particular 0.88, 0.83 and 0.92, respectively. Furthermore, TRPL measurements were taken on the same TDBA-DI:DBFPO films deposited on the different HTMs and on glass substrate (Fig. 4c and Supplementary Table 3). The results indicated comparable degree of exciton confinement in both EML/T2fQ and EML/DCDPA bilayers. Hole and electron mobilities of the HTM and ETM interlayers were further estimated with time-of-flight (TOF) measurements (Fig. 4d, e). It is seen that fluorine substitution in thiophene (T2fQ) instead of quinoxaline (TQ2f) highly improves the hole mobility of T2fQ to nearly 7.5 × 10^−3^ cm^2^ (V s)^−1^, which is fivefold higher relative to that of DCDPA and one order of magnitude above that of TQ2f. The electron mobility measured for TSPO1 was nearly four orders of magnitude lower, in accordance to the results published by others^41^. As a result, highly unbalanced hole and electron currents are expected, which, however, is not the case for T2fQ-based devices where nearly identical electron and hole currents were obtained in electron-only and hole-only (HOD) devices, respectively (Fig. 4f). On the contrary, highly unbalanced hole/electron currents were measured in the T2fQ-based HODs (Supplementary Fig. 22), which normally deteriorates the device performance.

Based on our findings to explain the highly balanced transport and superior performance of T2fQ-based OLEDs, we propose a device operation mechanism that involves the following: (i) highly efficient hole transport through T2fQ upon the application of an external bias, (ii) effective blocking of holes by the deep HOMO of TSPO1, (iii) electric field redistribution across TSPO1 and acceleration of electron transport therein due to built-up of holes at the EML/TSPO1 interface, (iv) exciton formation at the EML/TSPO1 interface and confinement by the interface exciplex, and (v) exciton transfer to the emitter and emission of blue light. This concept is well supported by the obtained data and is illustrated in Fig. 4g–i, which shows the electric fields (in the term of energy bands bending) in an ITO/NPD/T2fQ/DBFPO:TDBA-DI/TSPO1/LiF/Al device upon the application of forward bias and the different steps of device mechanism proposed above. Exciton confinement by the interface exciplex prohibits their diffusion across the 30 nm-thick EML, where a high probability of reaching the low energy levels of T2fQ exists, especially for the triplet excitons having diffusion lengths in the range of 15–30 nm^42,43^. Upon the formation of an interface exciplex, even though the intermolecular charge transfer between donor:acceptor and acceptor constituents is not as high as 100%, effective Förster resonant energy transfer results in excitons being transferred to the blue emitter (Fig. 4i).

To further support interface exciplex formation, we fabricated OLEDs with low emitter concentration doping in the EML. These devices exhibited a pronounced blue shift in their EL emission upon decreasing the emitter concentration (Supplementary Fig. 23). This is due to the exciplex emission and indicates suppressed energy transfer towards the emitter at lower doping levels. To unambiguously demonstrate the interface exciplex originated high-performance enhancement in our OLEDs, we inserted a 10 nm-thick DBFPO to serve as a buffer layer between the EML and TSPO1. By decreasing the concentration doping of the TDBA-DI emitter in the DBFPO host, besides a moderate decrease in EL intensity, attributed to suppressed energy transfer from DBFPO host to the emitter, non-profound EL shift was evident. Moreover, a significant reduction in the device performance was obtained through increasing the DBFPO buffer layer thickness from 0 to 10 nm (Supplementary Table 4), clearly demonstrating the critical role of interface exciplex formed between DBFPO and TSPO1 constituents in the device performance. In addition, the exciplex emission is clearly seen in the EL spectra taken at 2.5 V compared to those taken at 3.0 V forward bias (Supplementary Fig. 24). This indicates that the below-bandgap EL is due to CT excitons from interface exciplex, in accordance with previous studies^39^.

### OLED lifetime measurements

**Fig. 5:**
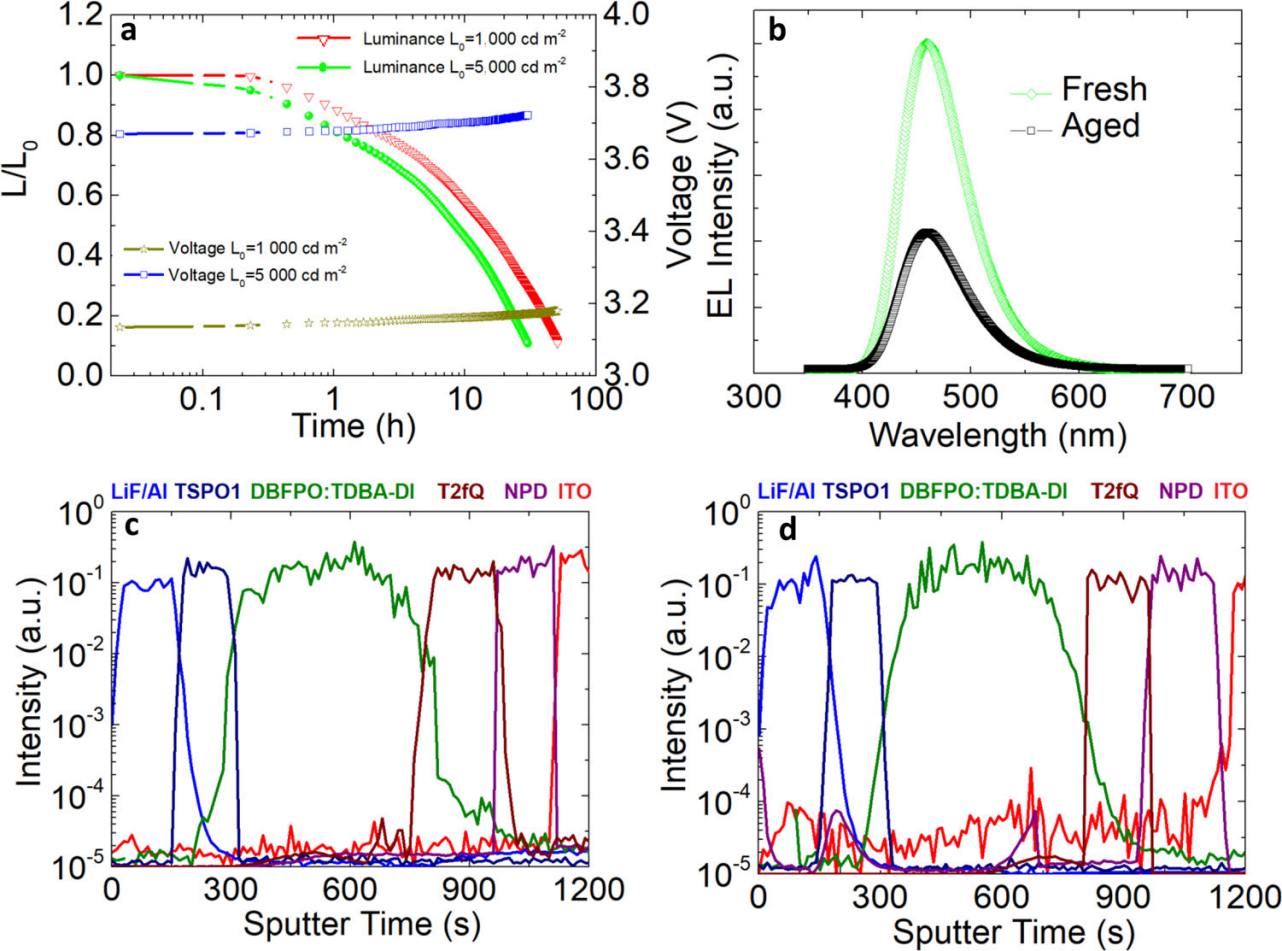
Lifetime measurements. **a** Variation of normalized luminance vs. time and driving voltage vs. time for initial luminance of 1000 or 5000 cd m^−2^. **b** Electroluminescence spectra of the fresh and degraded device. TOF-SIMS profiles of the **c** fresh and **d** degraded OLED stack: the *x*-axis represents the materials depth distribution starting with the cathode interface from data point 0 and reaching to the ITO anode electrode.

Besides high efficiencies and low turn-on voltage, the operational lifetime of an OLED is crucial for practical applications. Significant improvement in half-lifetime (T50) (up to 14 h for initial luminance of 1000 cd m^−2^) was obtained in our OLEDs, using not only T2fQ but also DCDPA and TQ2f interlayers, compared to the recently published OLEDs with the same emitter (i.e., 2 h)^19^ (Fig. 5a and Supplementary Fig. 25), indicating decreased charge carrier accumulation at the DBFPO/TSPO1 interface, which would be the origin of severe degradation^44^. The formation of the interface exciplex, however, strongly confines and rapidly transfers excitons towards the emitter. The excited host molecule instability-induced degradation, which was recently proposed by Adachi and colleagues^44^ as a major cause of emission loss in TADF OLEDs, is largely alleviated in our devices. Moreover, a minor change in the driving voltage was obtained even for a high luminance of 5000 cd m^−2^ (Fig. 5a), thus protecting our device from Joule heating and subsequent degradation^45^. The shape of the degraded EL spectra was in all cases similar to the initial ones (Fig. 5b and Supplementary Figs. 26 and 27), indicating that the degradation of those devices does not involve any changes in the recombination zone. This is in agreement with our proposed mechanism, which involves strong confinement of excitons within the DBFPO/TSPO1 interface and direct transfer towards the emitter^46^. Moreover, TOF secondary ion mass spectrometry (TOF-SIMS) measurements taken in the optimized OLED structures before and after degradation (Fig. 5c, d) revealed well-defined interfaces in the profiles, indicating strong resistance of the selected materials in degradation factors^47^. However, a large tail of the ITO profile extended within the whole device and a significant reduction of its intensity at the interface with NPD after degradation suggest that the primary degradation mechanism in these OLEDs derives through decomposition and diffusion of ITO electrode within the device, which has also been considered responsible for many types of OLED degradation^48,49^.

## Discussion

We fabricated blue TADF OLEDs using a low E_T_ HTM, a fluorinated TQ copolymer, which was designed with a high hole mobility of 7.5 × 10^−3^ cm^2^ (V s)^−1^. We combined this material with an interface exciplex formed between an ambipolar host and an ETM at the cathode device side. The HTM not only facilitated fast hole transfer towards the EML but also improved electron injection upon electric field redistribution within the low-mobility ETM. Interface exciplex strongly confined the generated excitons at the host/ETM interface, thus preventing leakage towards the exciton quenching HTM. These excitons were directly transferred to the blue emitter, thus suppressing charge and energy losses. Below-bandgap EL, highly improved efficiencies, suppressed efficiency roll-off and prolonged device lifetime were obtained, thus realizing the potential of using alternative interlayer materials and device architectures instead of well-designed emitters for further advancements in solution-processed LEDs.

## Methods

### Synthesis of organic materials

Hole injection material NPD (98% purity) and ETM TSPO1 (>99% purity) were purchased from Lumtec and Ossila, respectively, and were used as received. TDBA-DI and DCDPA were synthesized according to ref. ^19^. DBFPO was synthesized using a modified previously published procedure (see Supplementary Note 2). The syntheses of 5,8-dibromo-2,3-bis(3-(octyloxy)phenyl)quinoxaline,5,8-dibromo-6,7-difluoro-2,3-bis(3-(octyloxy)phenyl) quinoxaline and bis(trimethylstannyl)thiophene were performed according to already published procedures^50^. Then, (3,4-difluorothiophene-2,5-diyl)bis(trimethylstannane) was purchased from Derthon Optoelectronic Materials Science Technology Co., Ltd. The experimental condition for the polymerizations is analytically described below: the 5,8-dibromo-2,3-bis(3-(octyloxy)phenyl)quinoxaline (0.5 mmol) was combined with (3,4-difluorothiophene-2,5-diyl)bis(trimethylstannane) (0.5 mmol) for the T2fQ and the 5,8-dibromo-6,7-difluoro-2,3-bis(3-(octyloxy)phenyl)quinoxaline (0.5 mmol) was combined with the bis(trimethylstannyl)thiophene (0.5 mmol) for the TQ2f. Then, distilled toluene (0.025 M) was added at the reaction mixtures. Finally, tris(dibenzylideneacetone)dipalladium(0) [Pd_2_(dba_3_)] (0.02 equiv.) and tri(o-tolyl)phosphine [P(o-tol)_3_] (0.08 equiv.) were added, and the reaction mixture was stirred at 120 °C under argon atmosphere for 24 h. The polymers were purified by precipitation in methanol, filtered, and washed using a Soxhlet apparatus with methanol, acetone, hexane, and chloroform. The chloroform fractions were evaporated under reduced pressure and the polymers were precipitated in methanol, filtered through 0.45 mm PTFE filter and finally dried under high vacuum, rendering a dark brown solid with metallic appearance. The yields of the resulting polymers are the following: T2fQ = 93% and TQ2f = 91%. All reactions are air and light sensitive, and, therefore, were performed under argon and in the dark. All glasswares were washed using detergent (Teepol), rinsed with excess water, acetone, and methylene dichloride, and dried in an oven at 120 °C. All solvents and reagents were purchased from Aldrich. Toluene was distilled using calcium hydride (CaH_2_) and benzophenone prior to polymerization.

### Device fabrication and measurement of EL characteristics

The device structure was as follows: ITO/NPD (40 nm)/DCDPA or TQ2f or T2fQ (15 nm)/DBFPO 20% doped with TDBA-DI (30 nm)/TSPO1 (30 nm)/LiF (1 nm)/Al (100 nm). Prior to an OLED fabrication, cleaned glass substrates were patterned with a 250 nm-thick ITO anode deposited through a shadow mask using RF magnetron sputtering in an inert argon environment at a pressure of 1 × 10^−6^ mbar at a rate of 0.06 Å s^−1^. The substrates were heated to ~250 °C during deposition to increase the ITO conductivity. ITO-patterned glass substrates were sonicated in acetone, methanol, and 2-propanol for 15 min each and later dried in an oven. After that, the ITO substrates were treated with UV-ozone followed by organic layers deposition. NPD, DCDPA, DBFPO:TDBA-DI and TSPO1 were thermally deposited on the substrate in an inert chamber under a pressure of <4 × 10^−4^ Pa. The deposition rates of organic layers were 0.1–0.2 nm s^−1^. TQ2f and T2Qf were spin coated from THF solutions at 4000 r.p.m. for 45 s. The solution concentration was 10 mg mL^−1^. The solution concentration and spin rotation were optimized by performing several experiments. The deposition rate of LiF layer was 0.005–0.01 nm s^−1^, whereas for Al layer was 0.3–0.4 nm s^−1^. After the deposition of all layers, the OLED devices were encapsulated with a capping glass in an evaporation chamber filled with nitrogen. The OLED characteristics of all fabricated devices were recorded at RT in an air atmosphere using Agilent 4156C semiconductor parameter analyzer to monitor the electrical characteristics. The current density–voltage (*J*−*V*), luminance–voltage (*L*−*V*), and EL spectra were measured using a Konica Minolta CS-100A luminance meter and a CS2000A spectrometer coupled with a Keithley 2635A voltage and current source meter. The EQE measurements were conducted using C9920-12, Hamamatsu Photonics, Japan, with a source meter 2400, Keithley, Japan. Time-resolved PL measurements of the EML were conducted using a Hamamatsu streak camera containing a photocathode sensitized from the visible spectrum to 1300 nm and operated in the synchroscan mode. The samples were excited with 100 fs-long optical pulses at 365 nm by the frequency-doubled output of a mode locked Ti:sapphire laser. The film thickness was measured using a Dektak AlphaStep Profiler.

### Photoluminescence measurements

Steady-state PL measurements of TQs, DBFPO, and TSPO1 films were carried out on a Horiba Fluorolog-3 spectrometer (FL 3–22, Horiba Jobin Yvon). Samples were excited at different wavelengths (404 nm for the TQs and 280 nm for TSPO1, DBFPO, and mixtures) with a slit width of 10 nm and the emitted photons were collected in front-face geometry. Time-correlated single-photon counting measurements were carried out on the DeltaFlex system (Horiba Scientific). For fluorescence measurements, samples were excited with a pulsed laser diode at 404 nm (NanoLED, pulse width < 200 ps). For phosphorescence measurements, samples were excited with a SpectraLED at 361 nm. The emitted photons were collected with a picosecond photon detector (PPD-900). The TRPL decay curves of the EML was recorded at a detection wavelength of 450 nm.

### Angular-dependent fluorescence

Thin films of DBFPO:TDBA-DI were deposited on pre-cleaned glass substrates by vapor deposition and encapsulate with a capping glass under nitrogen atmosphere. The sample was attached to a hemicylinder prism with matched refractive index oil, and an excitation continuous wave laser with a wavelength of 365 nm and a power of <20 mW was illuminated on the thin films. Through a 410 nm cut-off filter, a polarizer, and collimating lens, PL intensity in a transverse magnetic mode was detected by a calibrated multichannel spectrometer (PMA-12, Hamamatsu Photonics). The PL intensities were acquired in each out-of-plane angle from 0° (vertical to the substrate surface) to 90° (horizontal to the substrate surface) with a step of 2°. The collected radiation patterns were analyzed using a commercially available software (Setfos 4.0, Fluxim AG, Switzerland). The measurements were conducted in an inert nitrogen atmosphere to avoid photochemical degradation as well as PL quenching induced by a combination of oxygen and UV light. The measurements were repeated on different samples to ensure reproducible and consistent results. The dipole orientation of the TADF emitter in the host matrix was determined from the analysis of the angle-dependent PL spectra. The spectra were resolved by using a *p*-polarizing filter and were measured by a fiber optical spectrometer. The angle-dependent *p*-polarized emission intensity at the peak wavelength of the PL spectrum of the emitting layer was detected. The emitting dipole orientation (i.e., the horizontal dipole ratio Θ_//_) was then determined by least square fitting of the measured angle-dependent *p*-polarized emission intensity with calculated results.

### Simulations

The theoretical limit of the device performance was performed using optical simulations based on the plane wave decomposition and as demonstrated by the scattering matrix formalism. Our simulations allowed us to calculate the maximum possible EQE, depending on the film thickness and refractive index in the stack layout by assuming balanced charge carrier injection and a singlet–triplet ratio of unity.

### TOF-SIMS measurements

A TOF-SIMS V spectrometer was utilized to depth profile the organic materials and completed devices. Analysis was completed utilizing a three-lens 30 kV BiMn primary-ion gun and the Bi^+^ primary-ion beam (operated in bunched mode, 10 ns pulse width, analysis current 1.0 pA) was scanned over a 25 × 25 μm area. Depth profiling was accomplished with a 1 kV oxygen-ion sputter beam (10.8 nA sputter current) raster of 150 × 150 μm. During profiling, all the spectra were collected at or below a primary-ion dose density of 1 × 10^12^ ions cm^–2^ to remain at the static SIMS limit. The data are plotted with the intensity for each signal at each data point normalized to the total ion counts measured at that data point, which diminishes artifacts from a changing ion yield in different layers when profiling through completed devices.

### Gel permeation chromatography

Average molecular weights per number ($\bar{{{{{{\rm{M}}}}}}_{{{{{\rm{n}}}}}}}$) and polydispersity indices (Đ) were determined with GPC at 80 °C on a Shimadzu liquid chromatograph (LC-20AD) system consisting of a DGU-20A5R degassing unit, a SIL-20AC HT auto sampler, a CTO-20AC column oven, a SPD-20AV UV-Vis detector, and a RID-20A refractive index detector connected in series. The system contains a PL-GEL 10 μm guard column, two PL-GEL 10 μm Mixed-C columns, and THF as the eluent. The instrument was calibrated with narrow polystyrene standards with *M*_p_ ranging from 4730 g mol^−1^ to 3187000 g mol^−1^.

### Nuclear magnetic resonance

^1^H- and ^13^C-NMR measurements were carried out in solutions (1% w/v) of the copolymers using CDCl_3_ (Acros 99.6%) as the solvent and tetramethylsilane as the integral standard on a Varian 600 MHz NMR spectrometer at ambient temperature.

### Absorption spectrometry

UV-Vis absorption of the polymer solutions (concentration 10^−5^ M) and the fabricated films were recorded with a Shimadzu 1900 spectrometer.

### Cyclic voltammetry

Cyclic voltammetry was conducted on a VersaSTAT4 potentiostat galvanostat with platinum (Pt) disk, Pt wire, and Ag/AgNO_3_ electrode as the working electrode, counter electrode, and reference electrode, respectively, using a 0.1 M solution of tetrabutylammonium hexafluorophosphate (n-Bu_4_NPF_6_) in anhydrous acetonitrile at a potential scan rate of 50 mV s^−1^. Thin films of samples were deposited onto the Pt disk working electrode from a chloroform solution. The potential of Ag/AgNO_3_ reference electrode was internally calibrated by using the ferrocene/ferrocenium redox couple (Fc/Fc^+^). The electrochemical energy levels were estimated by using the empirical formula: ${{{{{{\rm{E}}}}}}}_{{{{{{\rm{LUMO}}}}}}}=-[4.80+({{{{{{{\rm{E}}}}}}}_{{{{{{\rm{onse}}}}}}}}^{{{{{{\rm{Ox}}}}}}}-{{{{{{\rm{Fc}}}}}}}^{1/2})]$.

#### Theoretical calculations

All calculations of the model compounds studied in this work have been performed using the Gaussian 09 software package. The alkyl side chains substituents anchored onto the quinoxaline have been replaced with methyl groups in the model compounds for our calculations. Although the presence of these long alkyl chains enhances the solubility of these polymers and affects the charge carrier mobility and photovoltaic behavior of the polymer, from a computational point of view their replacement with shorter chains does not affect their optoelectronic properties (HOMO, LUMO, and bandgap) and thus the optimized structures of the molecules. The ground-state geometry of each model compound has been determined by a full geometry optimization of its structural parameters using the density functional theory (DFT) calculations, upon energy minimization of all possible isomers. In this work, the DFT calculations were performed using the HSEH1PBE/6-311G(d,p) basis set. All calculations were performed taking into account that the system is under vacuum conditions. No symmetry constraints were imposed during the optimization process. The geometry optimizations have been performed with a tight threshold that corresponds to root mean square residual forces smaller than 10^−5^ au for the optimal geometry. The energy level of the E_HOMO_ and the E_LUMO_ of the repetitive units of each polymer were carried out by using the same set of calculations. DFT/HSEH1PBE/6-311G has been found to be an accurate formalism for calculating the structural and electronic properties of many molecular systems. In our studies, the theoretical calculations were performed on dimer model compounds. The visualization of the molecular orbitals has been performed using GaussView 5.0.

## Supplementary information

Supplementary Information

## Data Availability

The data that support the findings of this study are available on request from the corresponding authors (M.V., A.R.b.M.Y and N.G.).
