# Supplementary material for: High efficiency blue organic light-emitting diodes with below-bandgap electroluminescence
Source: Nat Commun. 2021 Aug 11;12:4868. doi: 10.1038/s41467-021-25135-z (PMC8357948; doi:10.1038/s41467-021-25135-z)
Supplement: Supplementary file 1 — Supplementary Information [file 41467_2021_25135_MOESM1_ESM.pdf]

Supplementary Information

High efficiency blue organic light-emitting diodes with below-bandgap electroluminescence

Maria Vasilopoulou[1,*], Abd. Rashid bin Mohd Yusoff[2,*], Matyas Daboczi[3], Julio Conforto[4], Anderson Emanuel Ximim Gavim[4], Wilson Jose da Silva[4], Andreia Gerniski Macedo[4], Anastasia Soultati[1], George Pistolis[1], Fabio Kurt Schneider[4], Yifan Dong[5], Polina Jacoutot[5], Georgios Rotas[6], Jin Jang[7], Georgios C. Vougioukalakis[6], Christos L. Chochos[8,*], Ji-Seon Kim[3], Nicola Gasparini[5,*]

[1]Institute of Nanoscience and Nanotechnology, National Centre for Scientific Research Demokritos, Terma Patriarchou Grigoriou, 15341 Agia Paraskevi, Greece

[2]Department of Chemical Engineering, Pohang University of Science and Technology (POSTECH), Pohang, Gyeongbuk 37673, Republic of Korea

[3]Department of Physics and Centre for Processable Electronics, Imperial College London, London, SW7 2AZ, UK

[4]Universidade Tecnologica Federal do Parana, GPGEI – Av. Sete de Setembro, 3165 – CEP 80230-901, Curitiba, Parana, Brazil

[5]Department of Chemistry and Centre for Processable Electronics, Imperial College London, W120BZ, UK

[6]Department of Chemistry, National and Kapodistrian University of Athens, 15771 Athens, Greece

[7]Advanced Display Research Center, Department of Information Display, Kyung Hee University, Dongdaemoon-gu, Seoul 130-701, South Korea

[8]Institute of Chemical Biology, National Hellenic Research Foundation, 48 Vassileos Constantinou Avenue, Athens 11635, Greece

[†]These authors contributed equally to this work.

*Email: m.vasilopoulou@inn.demokritos.gr, abdrashid@postech.ac.kr, cchochos@advent-energy.com, n.gasparini@imperial.ac.uk

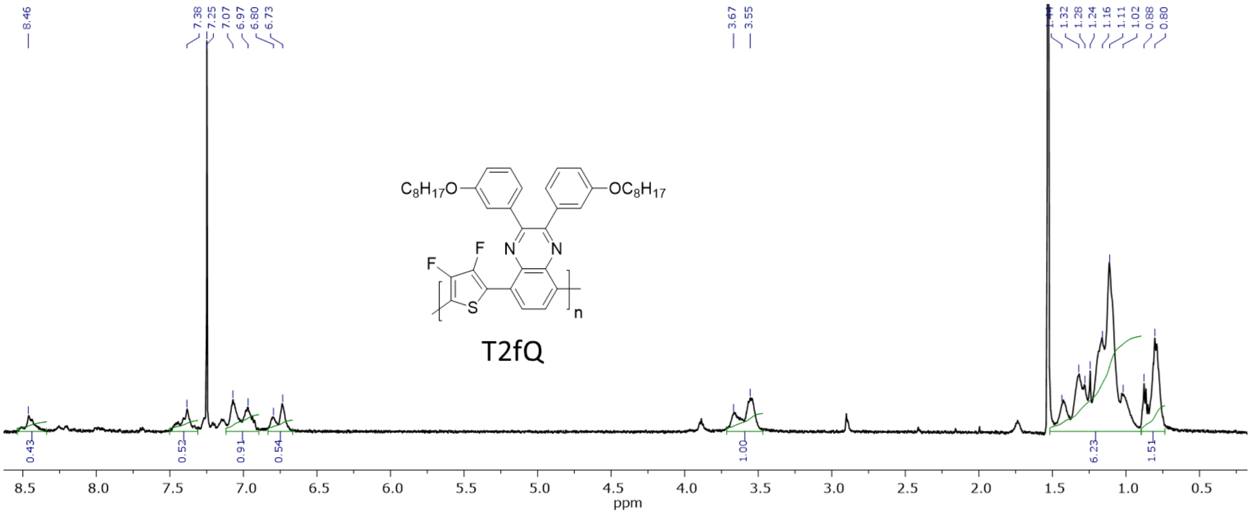

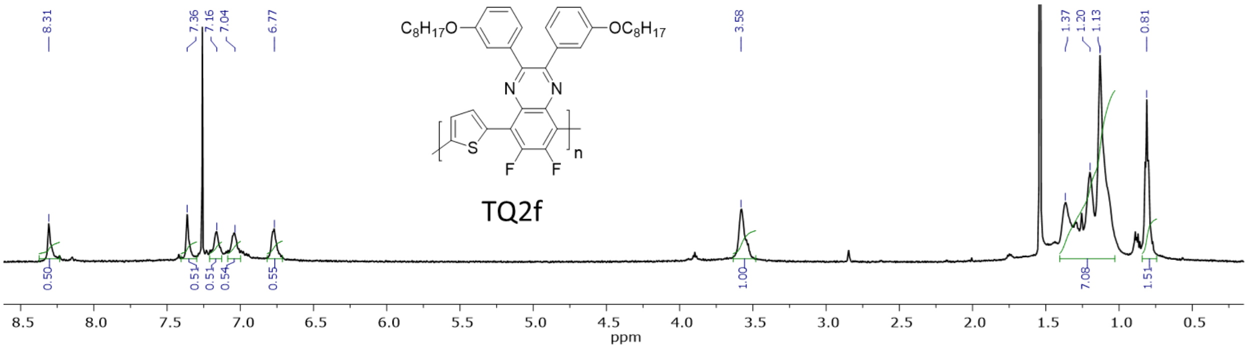

Supplementary Fig. 1 ^{1}H- (600 MHz) NMR spectra of T2fQ and TQ2f in $CDCl_3$.

T2fQ: ^{1}H-NMR ($CDCl_3$, 600 MHz) δ 8.46 (b, 1H), 7.38 (b, 1H), 7.07 - 6.97 (b, 2H), 6.80 - 6.73 (b, 1H), 3.67 - 3.55 (b, 2H), 1.44 - 1.02 (m, 14H), 0.88 - 0.80 (m, 3H).
TQ2f: ^{1}H-NMR ($CDCl_3$, 600 MHz) δ 8.31 (b, 1H), 7.36 (b, 1H), 7.16 (b, 1H), 7.04 (b, 1H), 6.77 (b, 1H), 3.58 (b, 2H), 1.37 - 1.13 (m, 14H), 0.81 (t, 3H).

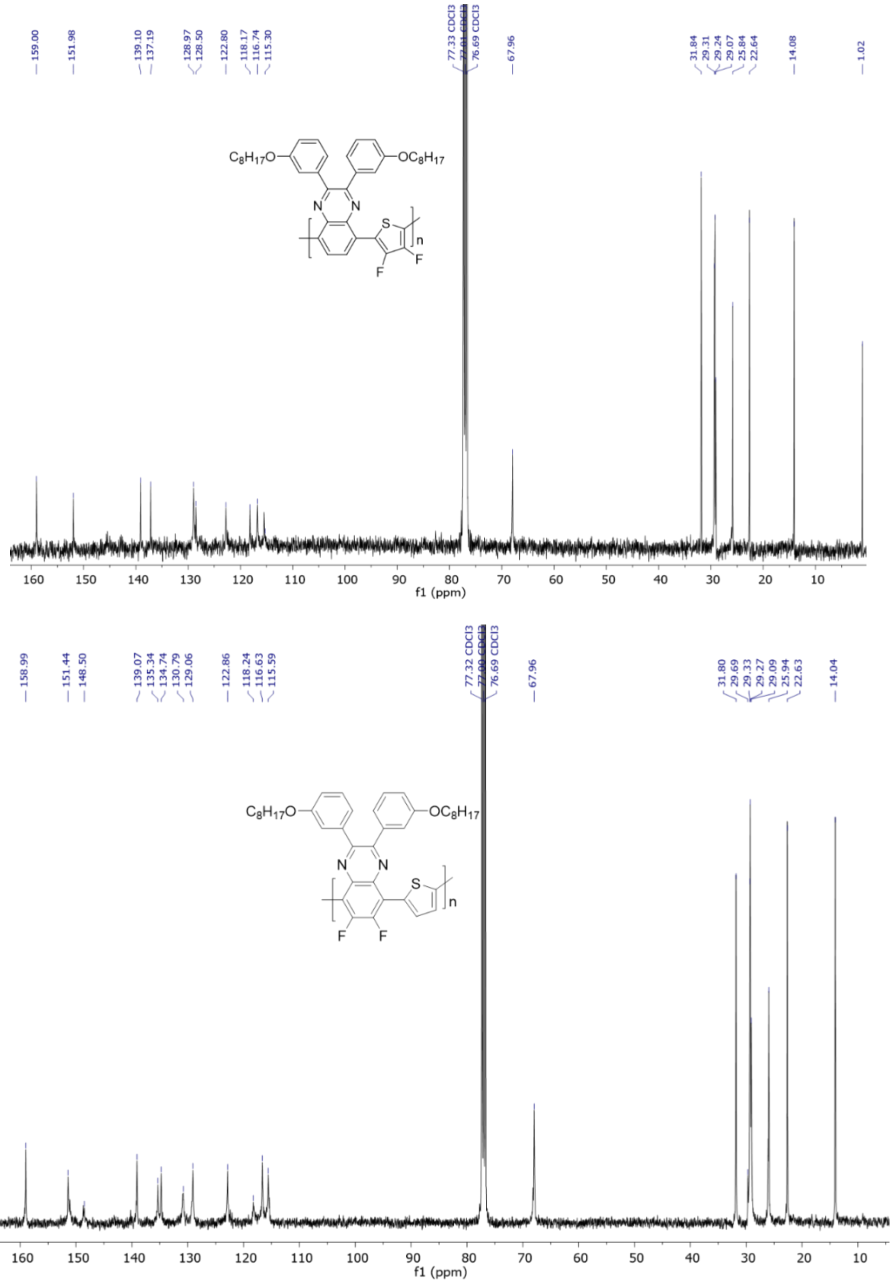

Supplementary Fig. 2 ^{13}C- (100 MHz) NMR spectra of T2fQ and TQ2f in $CDCl_3$.

T2fQ: ^{13}C-NMR ($CDCl_3$, 100 MHz) δ 159.00, 151.98, 139.10, 137.19, 128.97, 128.50, 122.80, 118.17, 116.74, 115.30, 67.96, 31.84, 29.31, 29.24, 29.07, 25.84, 22.64, 14.08, 1.02, SEC ($CDCl_3$): M_n = 6000 g/mol, PDI = 1.9, T_d = 225 ºC (5% loss)

TQ2f: ^{13}C-NMR ($CDCl_3$, 100 MHz) δ 158.99, 151.44, 148.50, 139.07, 135.34, 134.74, 130.79, 129.06, 122.86, 118.24, 116.63, 115.59, 67.96, 31.80, 29.69, 29.33, 29.27, 29.09, 25.94, 22.63, 14.04; SEC ($CDCl_3$): Mn = 22600 g/mol, PDI = 2.3, T_d = 225 ºC (5% loss).

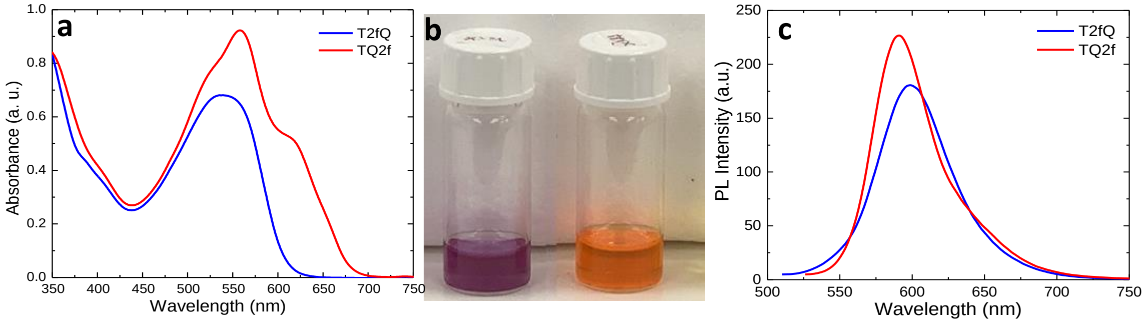

Supplementary Fig. 3 Optical properties of fluorinated TQs. a, Absorption spectra of TQ2f and T2fQ in tetrahydrofuran (THF) solutions and **b**, photographs of the corresponding solutions. **c**, Steady-state photoluminescence (PL) spectra of T2fQ and TQ2f in THF solutions.

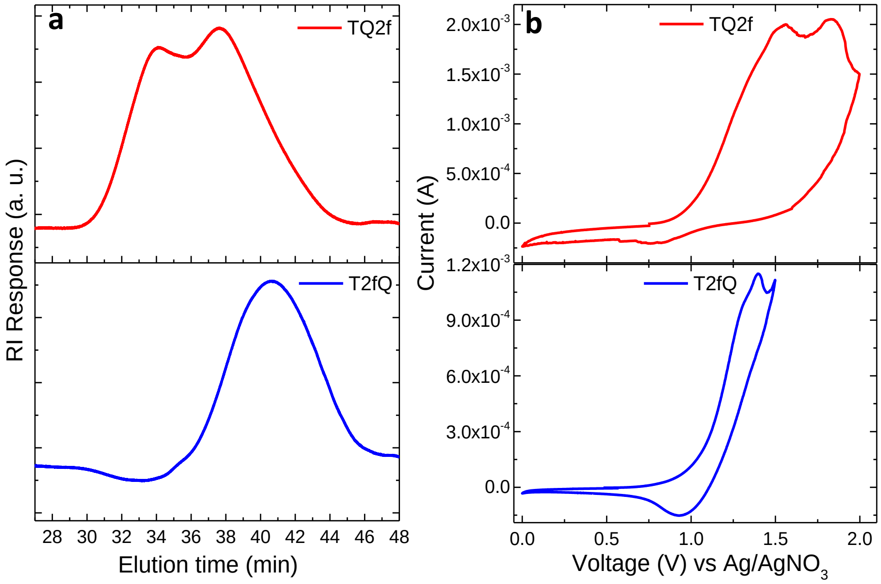

Supplementary Fig. 4 Electronic properties of fluorinated TQs[1-6]. a, Gel permeation chromatography (GPC) profiles of T2fQ and TQ2f. **b,** Cyclic voltammetry graphs of T2fQ and TQ2f during oxidation.

Supplementary Note 1. Calculation of the E_{HOMO} energy level based on the results of Supplementary Fig. 4:
$E_{ox}^{Ag/AgNO3}$ = +0.99 V (TQ2f).
$E_{ox}^{Ag/AgNO3}$ = +1.04 V (T2fQ).
$E_{ox}^{Fc/Fc+}$ = +0.99 – 0.09 = 0.90 V (TQ2f).
$E_{ox}^{Fc/Fc+}$ = +1.04 – 0.09 = 0.95 V (T2fQ).
TQ2f:E_{HOMO} = -(4.8 + 0.90) eV = -5.70 eV (TQ2f), E_g=1.82 eV, E_{LUMO}= (-5.70+1.82) eV= -3.88 eV.
T2fQ: E_{HOMO} = -(4.8 + 0.95) eV = -5.75 eV, E_g=2.04 eV, E_{LUMO}= (-5.75+2.04) eV= -3.71 eV.

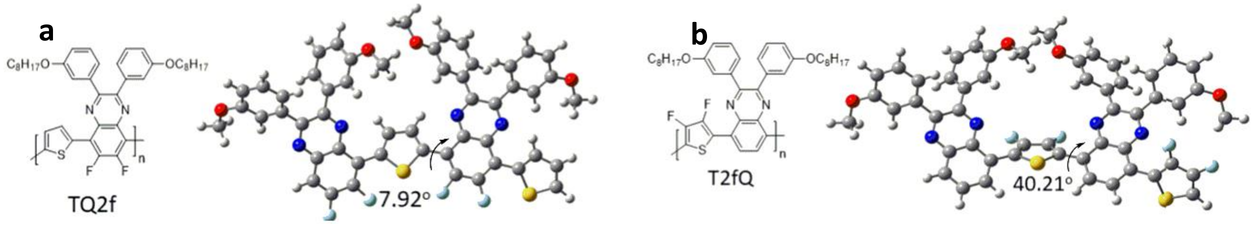

Supplementary Fig. 5 DFT calculations. [B3LYP/6-31G(d,p)] calculated dihedral angle for the dimer model compounds of **a**, TQ2f and **b**,TQ2f[7].

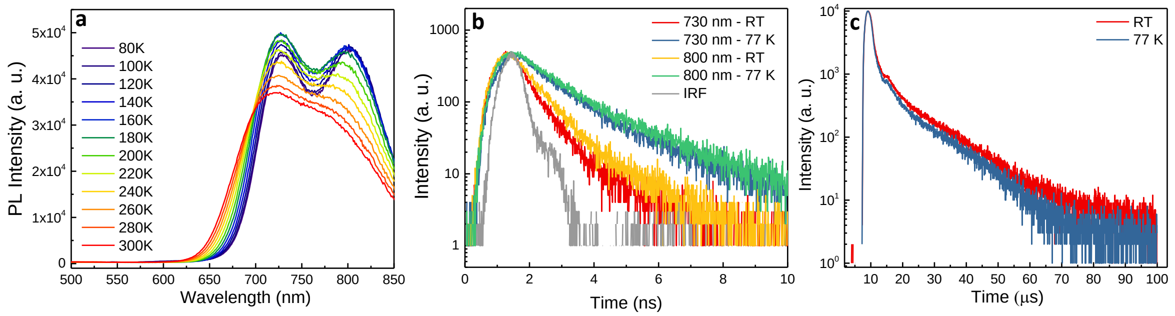

Supplementary Fig. 6 Photoluminescence spectra of TQ2f films. a, Steady-state PL spectra of TQ2f thin films deposited from THF solutions in quartz substrates taken at different temperatures ranging from room temperature (RT) to 80 K. **b**, Transient PL decay curves in the nanosecond scale of the same TQ2f films measured at RT and 77 K and at different detection wavelengths. **c**, Transient PL decay curves in the microsecond scale of the same films measured at RT and 77 K.

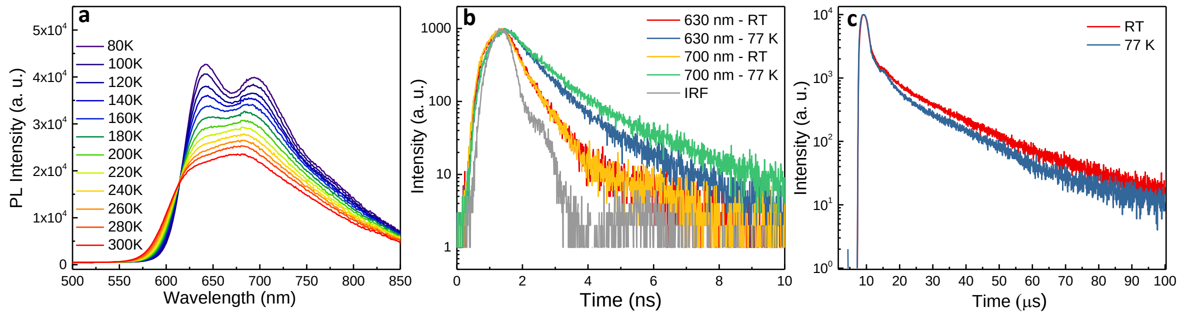

Supplementary Fig. 7 Photoluminescence spectra of T2fQ films. a, Steady-state PL spectra of T2fQ thin films deposited from THF solutions in quartz substrates taken at different temperatures ranging from room temperature (RT) to 80 K. **b**, Transient PL decay curves in the nanosecond scale of the same T2fQ films measured at RT and 77 K and at different detection wavelengths. **c**, Transient PL decay curves in the microsecond scale of the same films measured at RT and 77 K.

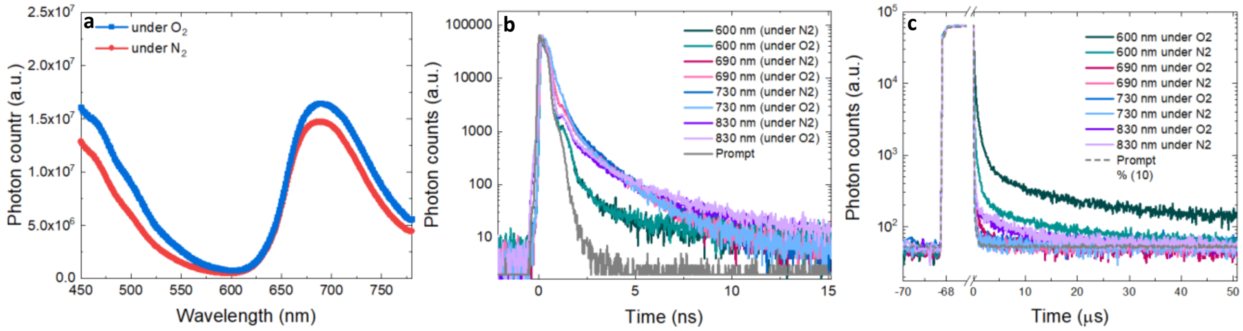

Supplementary Fig. 8 Photoluminescence study of TQ2f films. a, Steady-state PL spectra of TQ2f thin films deposited from THF solutions in quartz substrates taken at RT in oxygen (O_2) and nitrogen (N_2) environment. **b**, Transient PL decay curves in the nanosecond scale of the same TQ2f films measured at RT in O_2 and N_2 environment and at different detection wavelengths. **c**, Transient PL decay curves in the microsecond scale of the same films measured at RT in O_2 and N_2 environment and at different detection wavelengths.

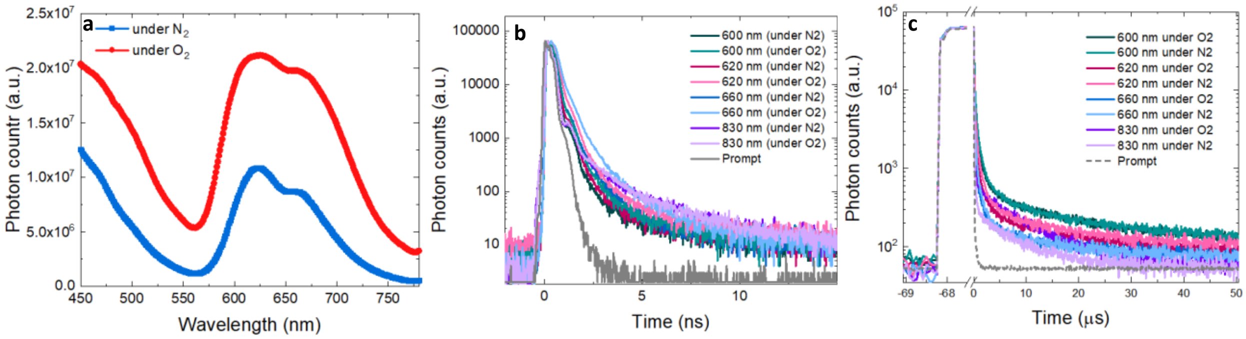

Supplementary Fig. 9 Photoluminescence study of T2fQ films. a, Steady-state PL spectra of T2fQ thin films deposited from THF solutions in quartz substrates taken at RT in oxygen (O_2) and nitrogen (N_2) environment. **b**, Transient PL decay curves in the nanosecond scale of the same T2fQ films measured at RT in O_2 and N_2 environment and at different detection wavelengths. **c**, Transient PL decay curves in the microsecond scale of the same films measured at RT in O_2 and N_2 environment and at different detection wavelengths.

Supplementary Note 2. Synthesis of bis(phosphinoxide) dibenzofuran 3 (DBFPO)[8].

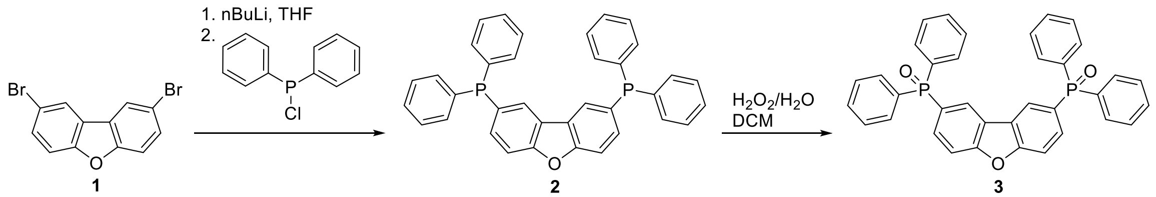

Synthesis of 2,8-bis(diphenylphosphanyl)dibenzo[*b*,*d*]furan 2. Butyllithium (2.5M in hexanes, 2.7 mL, 6.75 mmol) was added dropwise into a stirred suspension of 2,8-dibromodibenzofuran **1** (1 g, 3.07 mmol) in dry THF (16 mL) under argon, using a liquid N_2/ethanol slush bath so that the temperature remains at -110^oC. Then, reaction temperature was left to rise up to -70^oC (without removing the slush bath), before adding again liquid N_2 in the bath, bringing it back to -110^oC. This was repeated for 2.5 hours, during which a thick slurry is formed. Then, chlorodiphenylphosphine (1.24 mL, 6.75 mmol) was added dropwise, keeping the temperature below -80^oC. The mixture was stirred at -80 to -60^oC for 1 hour, during which the slurry dissolves. Then the reaction is left to reach room temperature and stirred for 18 additional hours. Water (1 mL) was added dropwise to the stirred mixture and the volatiles were evaporated. The residue was transferred to a separatory funnel with the aid of dichloromethane (50 mL) and brine (150 mL), the phases were separated and the aqueous layer was extracted with DCM (2x40 mL). The combined organic phases were washed with brine (2x40 mL), dried ($MgSO_4$) and the solvent was evaporated, leaving a highly viscous oil. This residue was purified with column chromatography (dry loading, silica gel, 5% ethyl acetate / petroleum ether), yielding **2** as white solid (888 mg, 54%). Rf (5% Ethyl Acetate / Petroleum Ether) = 0.4. ^{1}H NMR (400 MHz, $CDCl_3$) δ: 7.93 (d, J = 8.0 Hz, 1H, H-1), 7.57 (d, J = 8.4 Hz, 1H, H-8), 7.45 (t, J = 7.7 Hz, 1H, H-7), 7.39 – 7.34 (m, 10H, PhH). ^{13}C NMR (101 MHz, $CDCl_3$) δ: 157.12, 137.56 (d, J = 10.7 Hz), 133.67 (d, J = 19.3 Hz), 133.31 (d, J = 18.7 Hz), 131.36 (d, J = 11.2 Hz), 128.87, 128.69 (d, J = 6.9 Hz), 127.04 (d, J = 25.8 Hz), 124.42 (d, J = 9.8 Hz), 112.19 (d, J = 7.3 Hz). ^{31}P NMR (162 MHz, $CDCl_3$) δ: -5.00 ppm.

Synthesis of dibenzo[*b*,*d*]furan-2,8-diylbis(diphenylphosphine oxide) 3. Excess aqueous hydrogen peroxide (30%, 3 mL, 26.51 mmol) was added dropwise in a solution of **2** (600 mg, 1.118 mmol) in dichloromethane (30 mL) at -5^oC and the mixture was stirred vigorously at 0^oC for 1h. Water (20 mL) and DCM (20 mL) were then added, the phases were separated, the organic phase was washed with water (2x20 mL), dried ($MgSO_4$), and the solvent was evaporated. The residue is dissolved in 4-5mL DCM (a cloudy solution is formed), filtered through celite (cotton/pipette) and washed with DCM to yield a clear solution. The solvent was evaporated and the residue was triturated with diethyl ether, yielding **3** as bright white powder (573 mg, 90%). Rf (Ethyl Acetate) = 0.1. ^{1}H NMR (400 MHz, $CDCl_3$) δ: 8.28 (d, J = 11.9 Hz, 1H, H-1), 7.77 (ddd, J = 11.1, 8.6, 1.2 Hz, 1H, H-7), 7.68 – 7.63 (m, 5H, H, H-8, H-1',6'), 7.53 (td, J = 7.3, 1.1 Hz, 2H, H-4'), 7.44 (td, J = 7.5, 2.8 Hz, 4H, H-3', 5'). ^{13}C NMR (101 MHz, $CDCl_3$) δ: ^{13}C NMR (101 MHz, Chloroform-*d*) δ 158.58 (d, J = 2.9 Hz), 132.54 (d, J = 104.7 Hz), 132.20 (d, J = 2.9 Hz), 132.09 (d, J = 9.9 Hz), 131.73 (d, J = 11.8 Hz), 128.70 (d, J = 12.1 Hz), 127.73 (d, J = 105.4 Hz), 125.80 (d, J = 11.3 Hz), 123.92 (dd, J = 14.5, 1.1 Hz), 112.33 (d, J = 13.3 Hz). ^{31}P NMR (162 MHz, $CDCl_3$) δ: 29.34 (m) ppm.

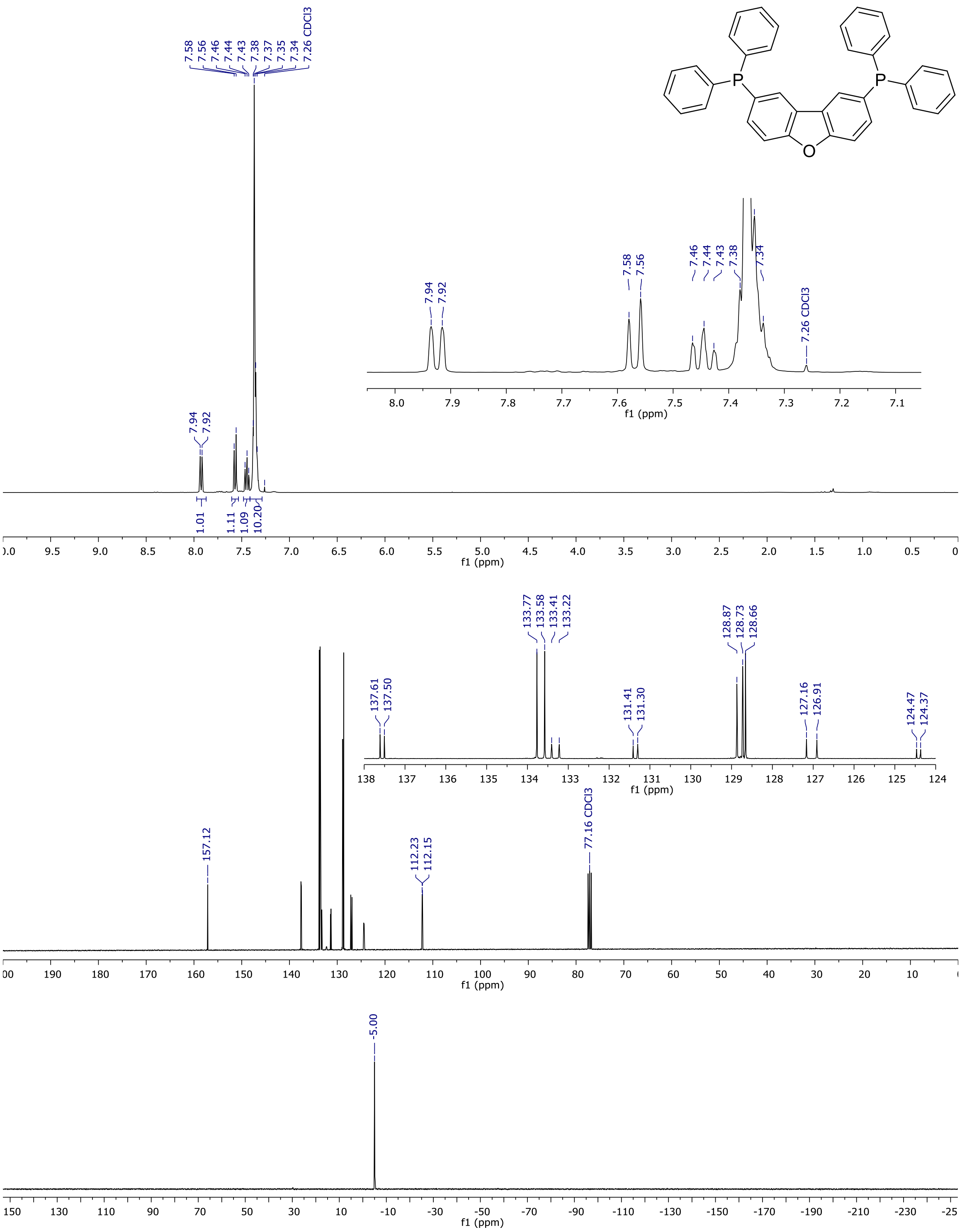

Supplementary Fig. 10 ^{1}H NMR (400 MHz, top), ^{13}C NMR (101 MHz, middle) and ^{31}P NMR (162 MHz, bottom) of **2** in $CDCl_3$.

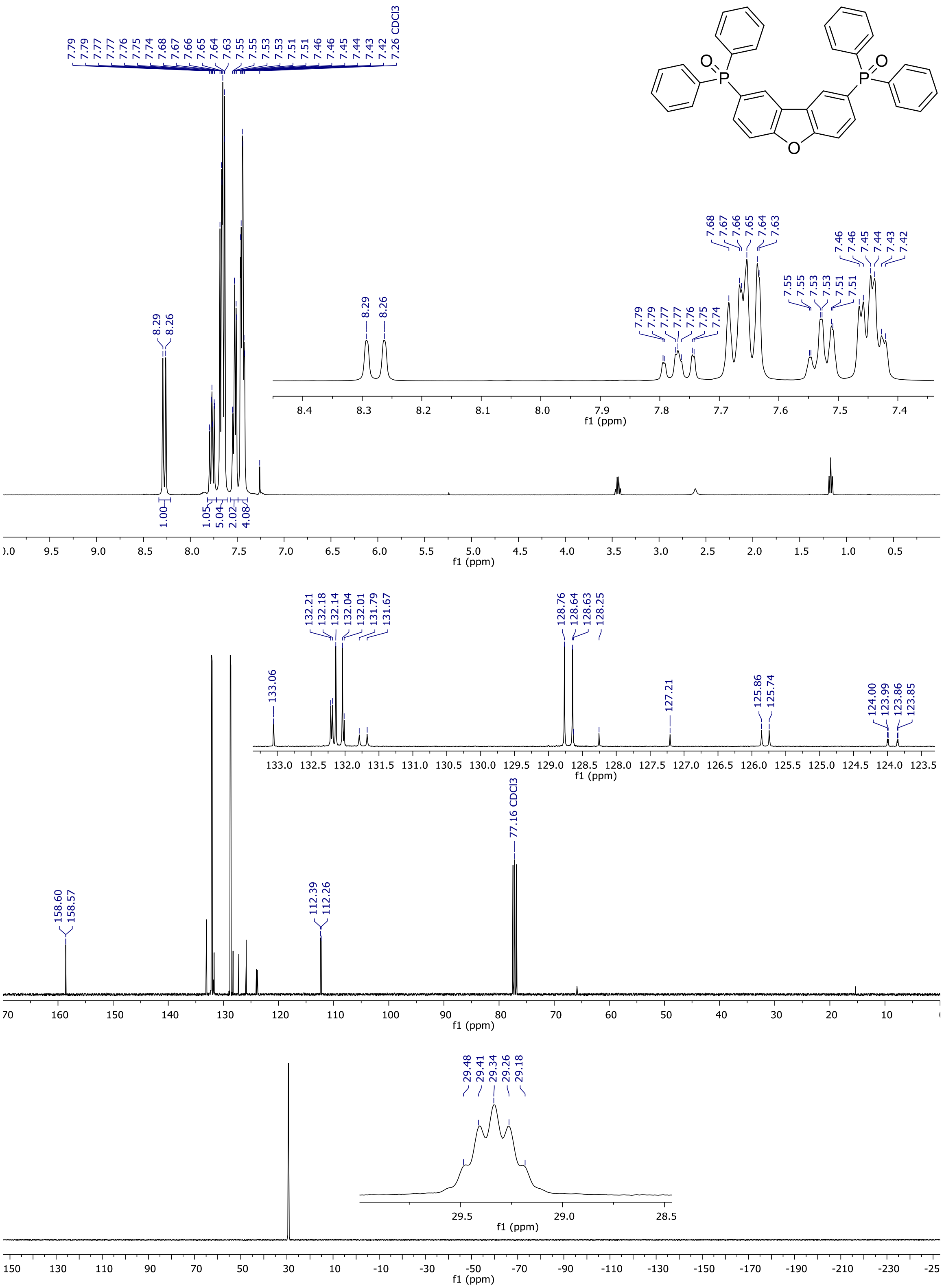

Supplementary Fig. 11 ^{1}H NMR (400 MHz, top), ^{13}C NMR (101 MHz, middle) and ^{31}P NMR (162 MHz, bottom) of **3** in $CDCl_3$.

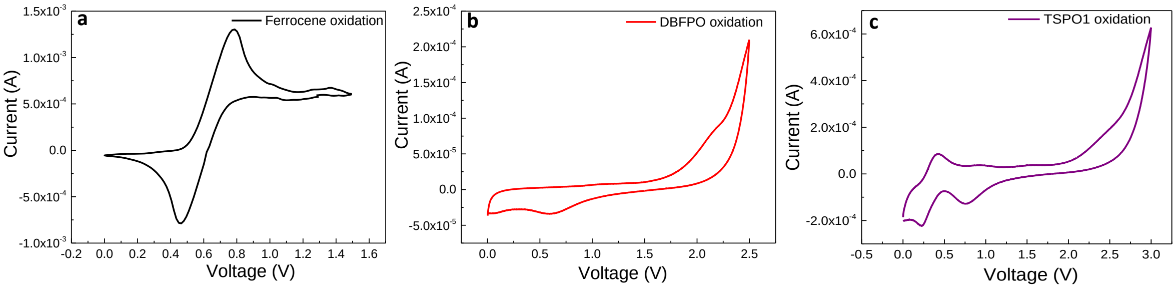

Supplementary Fig. 12 Electronic properties of DBFPO. Cyclic voltammetry graphs depicting the oxidation of **a**, ferrocene reference, **b**, DBFPO host and **c**, TSPO1 electron transport material.

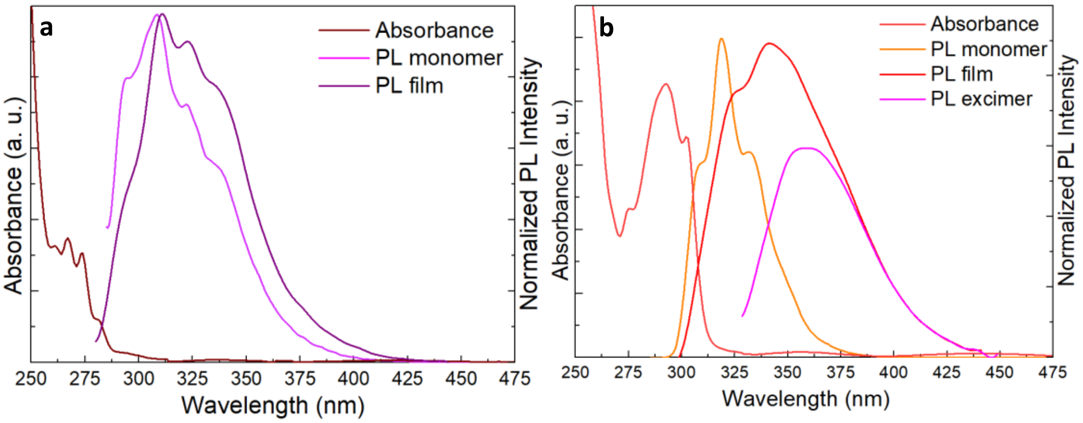

Supplementary Fig. 13 Optical properties of TSPO1 and DBFPO. a, UV-Vis absorption spectrum of TSPO1 (monomer in $5x10^{-7}$ M chloroform solution). Normalized PL spectra of TSPO1 monomer in solution and film. **b**, UV-Vis absorption spectrum of a DBFPO (monomer in solution). Normalized PL emission spectra of DBFPO monomer in solution and film. Through the subtraction between the DBFPO's monomer and film normalized PL spectra an excimer emission in the film centred at 361 nm was revealed. This excimer emission is fully consistent with the literature reports[8]. All PL spectra were obtained upon 280 nm excitation.

Supplementary Note 3. Calculations of HOMO, LUMO levels of DBFPO and TSPO1 based on Supplementary Figs. 12 and 13:

DBFPO: E_{ox} (Fc) = 0.62 V, E_{ox} (DBFPO) = 1.72 V, E_{ox} (DBFPO vs Fc) = 1.72 – 0.62 = 1.1 V
E_{HOMO} [DBFPO]= -(5.1 + E_{ox} (DBFPO vs Fc)) = -(5.1 + 1.1) = -6.2 eV
E_g (DBFPO) = 4.0 eV, E_{LUMO} [DBFPO] = -6.2 + 4.0 = -2.2 eV
TSPO1: E_{ox} (Fc) = 0.62 V, E_{ox} (TSPO1) = 2.12 V, E_{ox} (TSPO1 vs Fc) = 2.12 – 0.62 = 1.5 V
E_{HOMO} [TSPO1]= -(5.1 + E_{ox} (TSPO1 vs Fc)) = -(5.1 + 1.5) = -6.6 eV
E_g (TSPO1) = 4.2 eV, E_{LUMO} [TSPO1] = -6.6 +4.2 = -2.4 eV

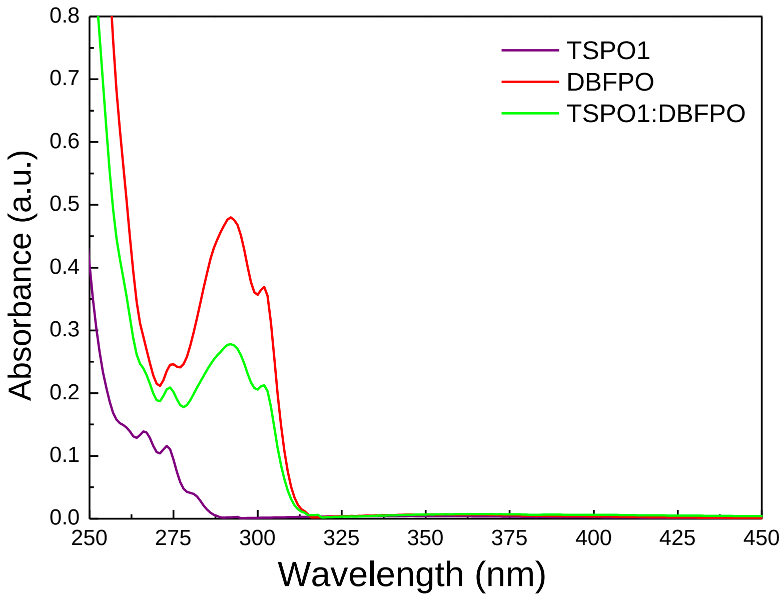

Supplementary Fig. 14 Absorption study. Absorption spectra of TSPO1, DBFPO and TSPO1:DBFPO 1:1 films as-deposited in air.

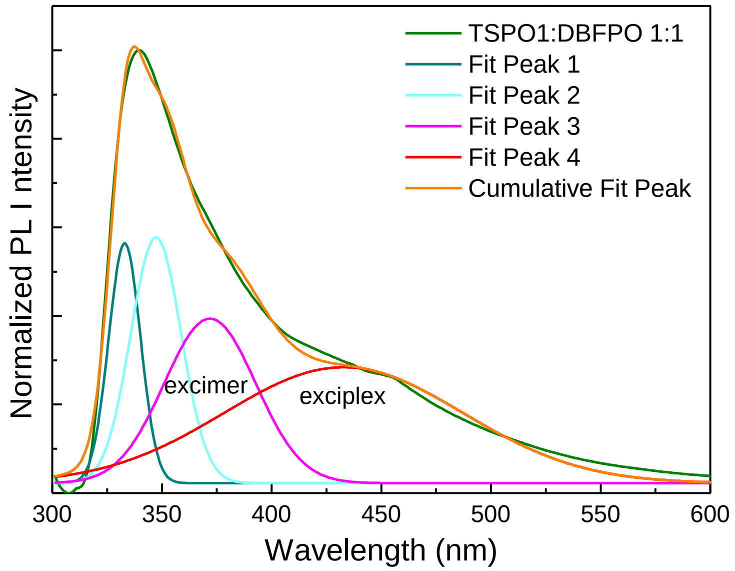

Supplementary Fig. 15 Photoluminescence peak fitting. Deconvolution of the TSPO1:DBFPO 1:1 film PL spectrum taken at 77 K using gaussian fit for exciplex identification. Besides the two peaks at 330 and 345 nm which are due to monomers emission, the excimer from DBFPO film (centered at 370 nm) is also evident. The broad peak centered at 440 nm is attributed to exciplex emission derived from intermolecular interactions between TSPO1 and DBFPO constituents.

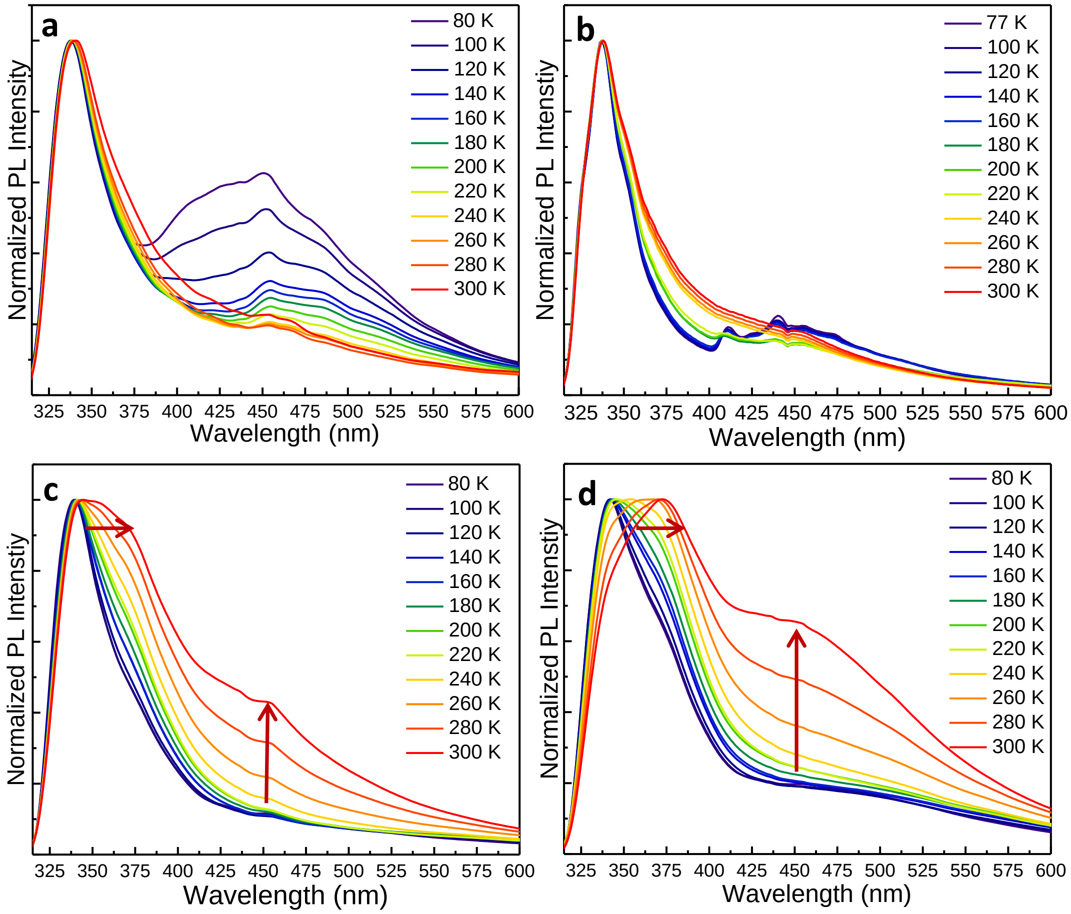

Supplementary Fig. 16 Photoluminescence study of TSPO1, DBFPO and mixtures. a, Normalized photoluminescence spectra of a TSPO1 film under different temperatures (starting from 80 K). The structured phosphorescence emission of TSPO1 at low temperature is evident. **b**, Normalized photoluminescence spectra of a TSPO1:DBFPO co-evaporated film (molecular ratio 10:1) at different temperatures (starting from 77 K). **c**, Normalized photoluminescence spectra of a TSPO:DBFPO co-evaporated film (molecular ratio 1:10) at different temperatures (starting from 80 K). **d**, Normalized photoluminescence spectra of a DBFPO film at different temperatures (starting from 80 K). All films were excited with 280 nm.

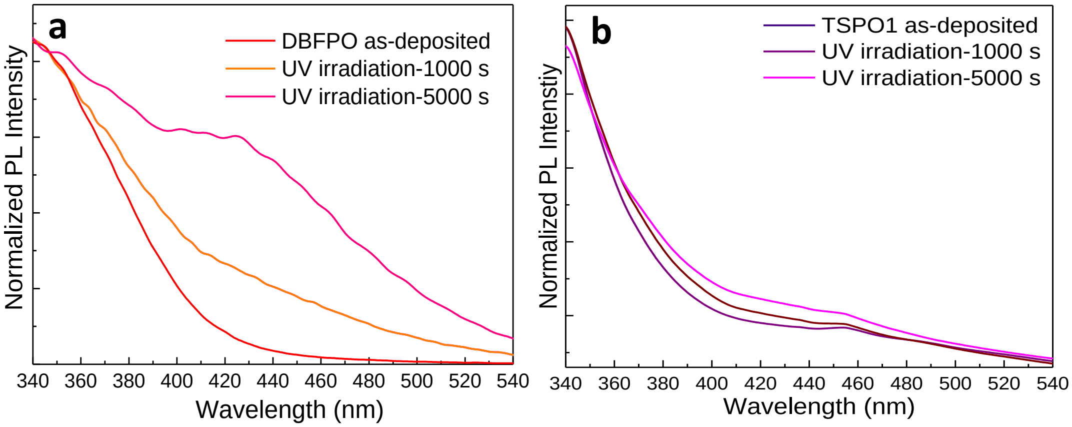

Supplementary Fig. 17 PL study under UV irradiation. PL spectra taken in a, DBFPO and b, TSPO1:DBFPO 1:1 films as-deposited and upon UV irradiation (in ambient air) for 1000 and 5000s. All films were excited at 280 nm.

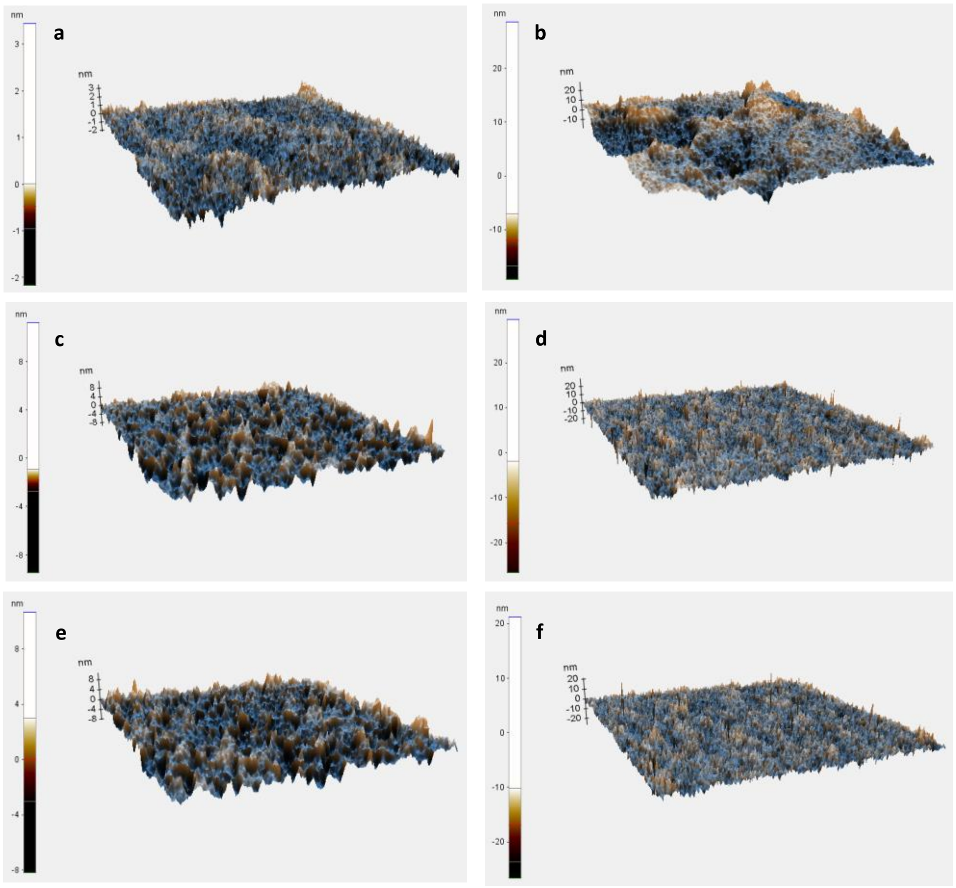

Supplementary Fig. 18 Film formation properties. Atomic force microscopy images of **a**, a DCDPA film, **b**, the EML on DCDPA, **c**, a TQ2f film, **d**, the EML on TQ2f, **e**, a T2fQ film and **f**, the EML on T2fQ. The root-mean-square (RMS) surface roughness was: DCDPA: 2.31nm, DCDPA/EML: 16.37nm, T2fQ: 4.29nm, T2fQ/EML: 10.37nm, TQ2f: 6.73nm, TQ2f/EML: 10.22nm).

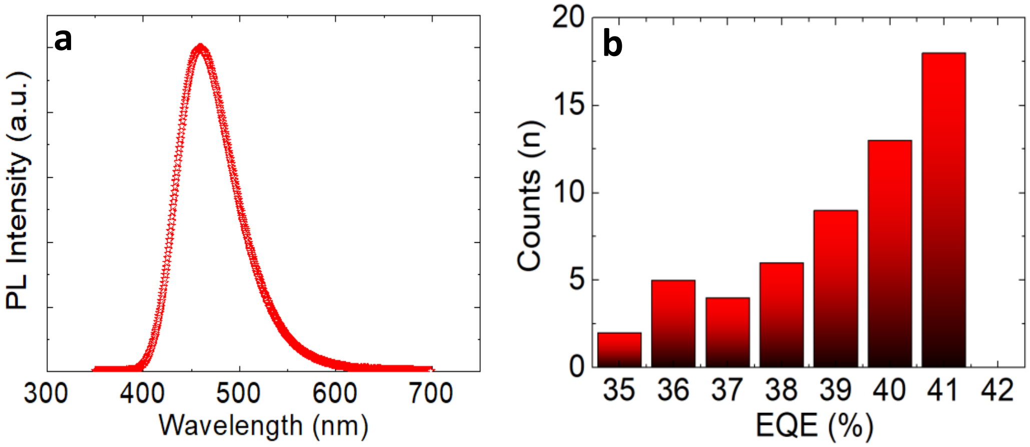

Supplementary Fig. 19 Additional T2fQ-based OLED data. a, Photoluminescence (PL) spectrum of the EML on top of the T2fQ on NPD/ITO and **b,** the histogram showing the variation of EQE in a batch of 56 independent devices. The extracted mean EQE is 34.62%.

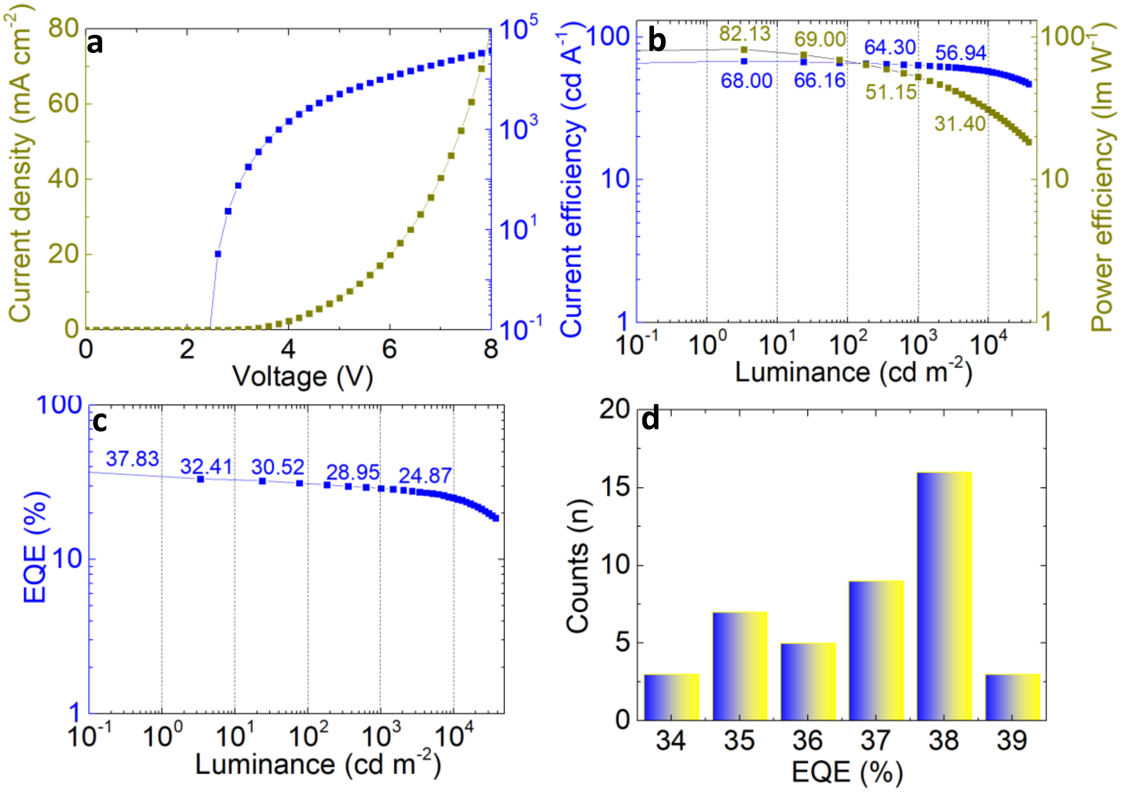

Supplementary Fig. 20 Performance metrics of OLEDs with DCDPA. a, Current density-luminance versus voltage (J-V-L) characteristics and **b**, current efficiency and power efficiency versus luminance of the blue TADF OLED with the DCDPA as the hole transport/electron blocking layer **(**HTL/EBL). **c,** EQE versus luminance and **d**, histogram of the obtained EQEs in a batch of 43 identical blue TADF OLEDs with the DCDPA HTL/EBL.

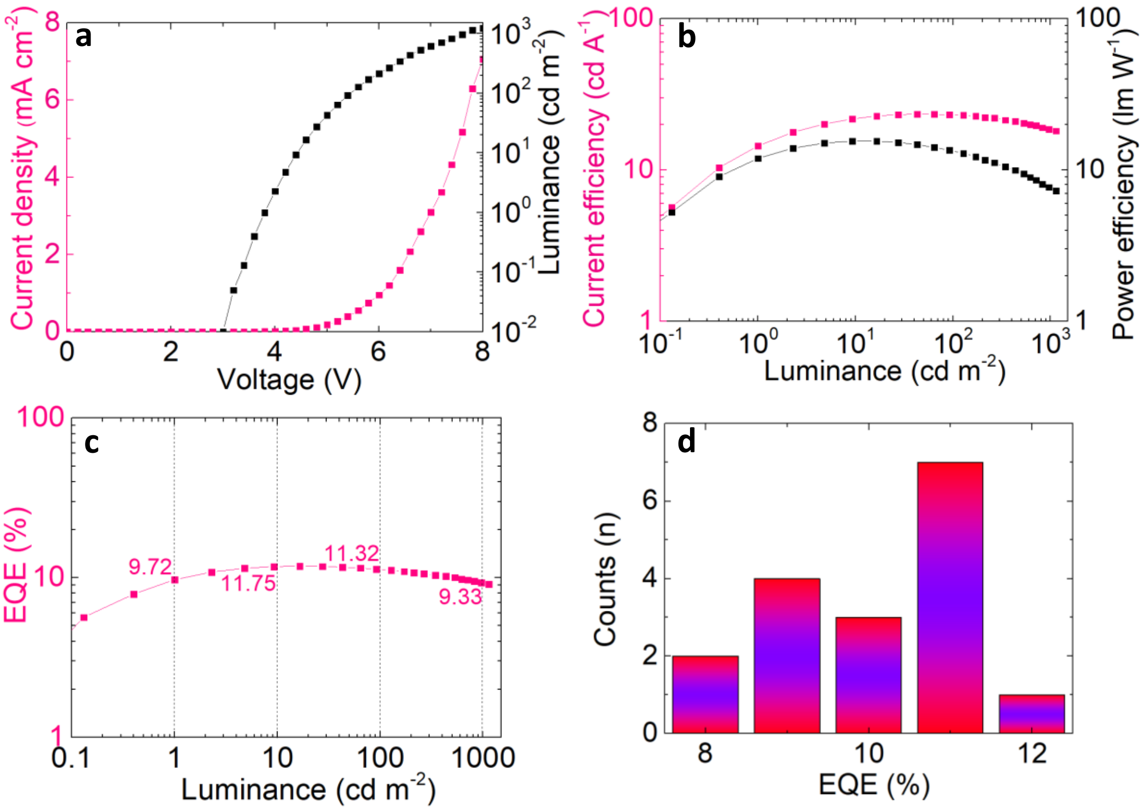

Supplementary Fig. 21 Performance metrics of blue TADF OLEDs based on TQ2f. **a**, Current density-luminance versus voltage (J-V-L) characteristics and **b**, current efficiency and power efficiency versus luminance of the best performing blue OLEDs using TQ2f. **c**, EQE versus luminance and **d**, histogram of the obtained EQEs in a batch of 17 identical blue TADF OLEDs with the TQ2f HTL/EBL material.

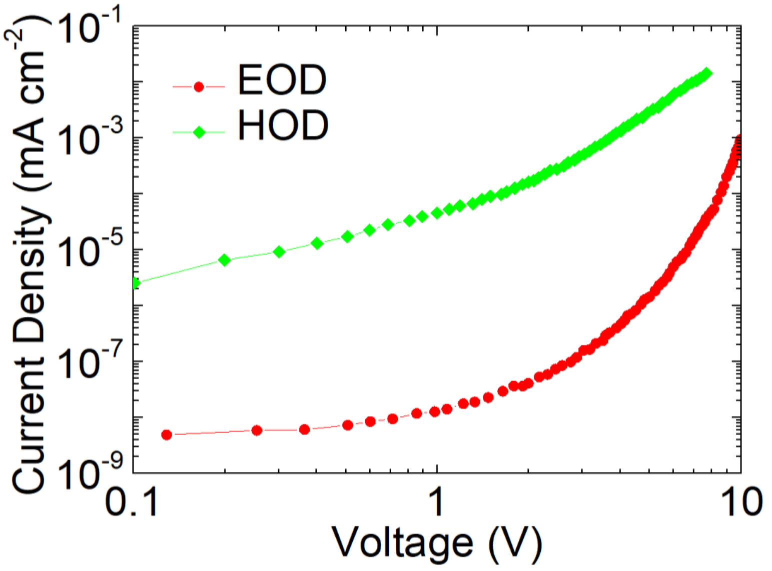

Supplementary Fig. 22 Electron and hole only currents. Current density-voltage characteristics taken in hole-only and electron-only devices using the low mobility TQ2f as hole transport interlayer.

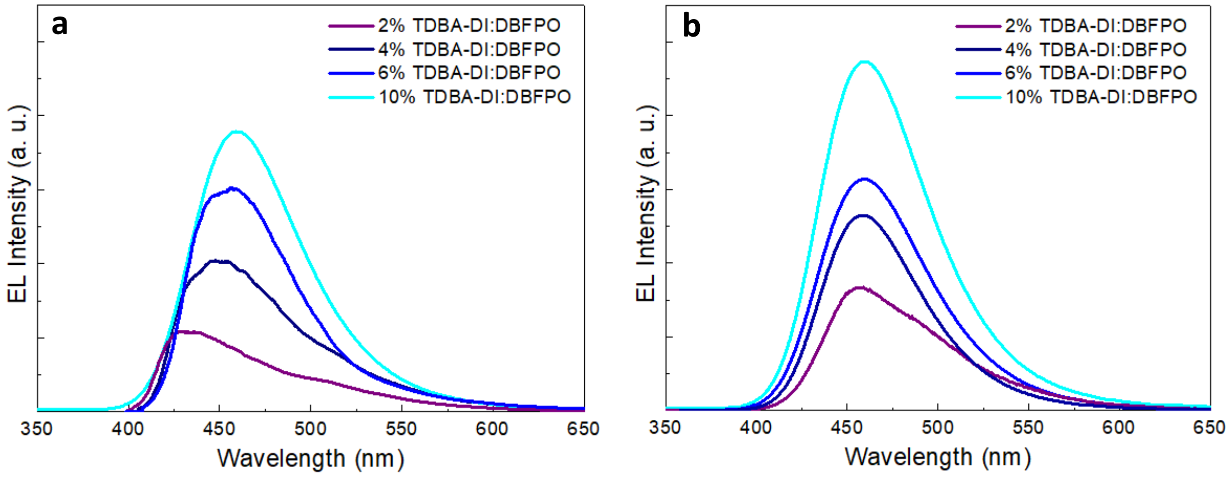

Supplementary Fig. 23 Electroluminescence study. **a**, EL spectra of OLEDs with the structure ITO/NPD (40 nm)/ T2fQ (15 nm)/TDBA-DI x%-doped in DBFPO (30 nm)/TSPO1 (30 nm)/LiF (1 nm)/Al (100 nm). **b**, EL spectra of OLEDs with the structure ITO/NPD (40 nm)/ T2fQ (15 nm)/TDBA-DI x%-doped in DBFPO (30 nm)/DBFPO (10nm)/TSPO1 (20 nm)/LiF (1 nm)/Al (100 nm). The EL spectra were measured at a constant driving voltage of 3.0 V.

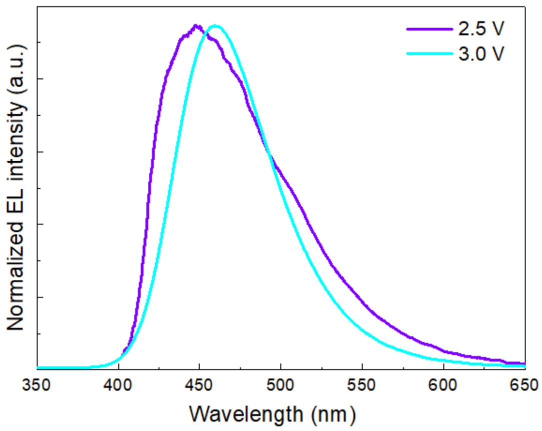

Supplementary Fig. 24 Origin of below-bandgap electroluminescence. Normalized EL spectra taken in OLEDs with the structure ITO/NPD (40 nm)/ T2fQ (15 nm)/TDBA-DI 10%-doped in DBFPO (30 nm)/TSPO1 (30 nm)/LiF (1 nm)/Al (100 nm) at 2.5 and 3.0 V forward bias.

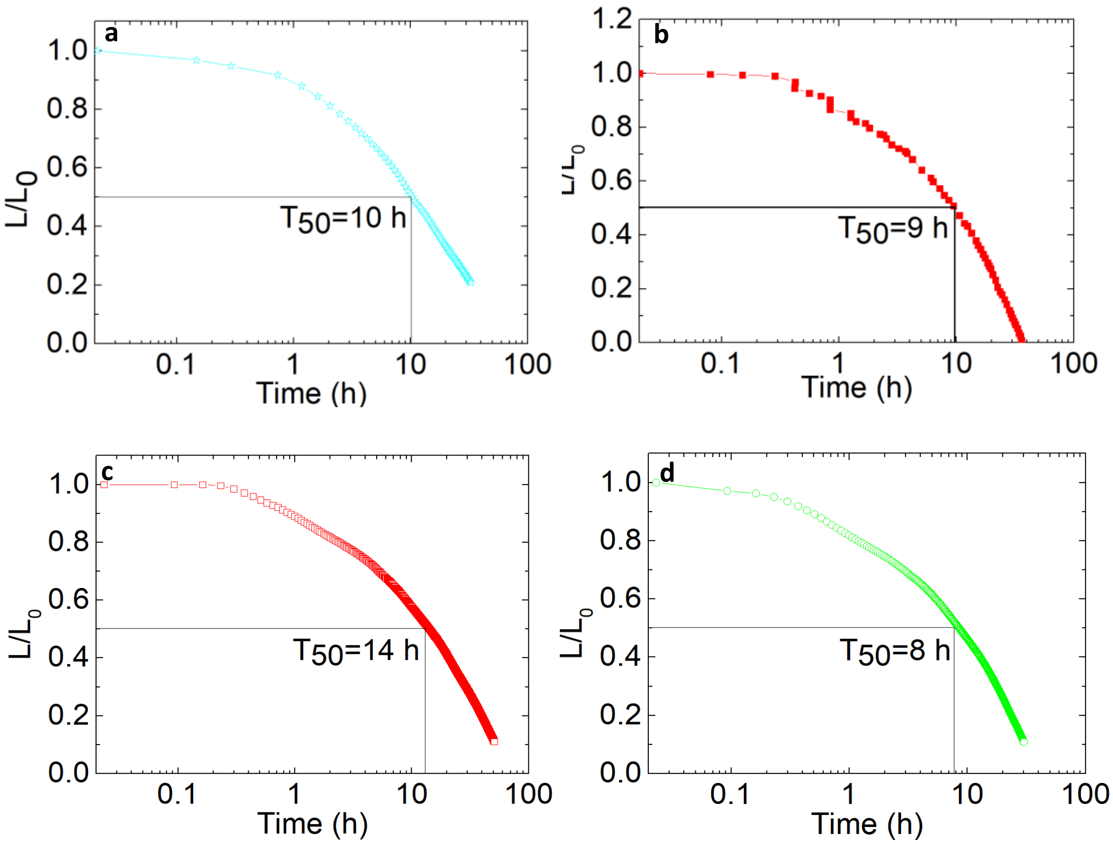

Supplementary Fig. 25 Degradation study. a, Half lifetime of OLEDs using DCDPA as hole transport material for an initial luminance of 1000 cd m^{-2}. **b**, Half lifetime of OLEDs using TQ2f as hole transport/electron blocking material for an initial luminance of 1000 cd m^{-2}. Half lifetime of OLEDs using T2fQ as hole transport/electron blocking material for an initial luminance of **c,** 1000 cd m^{-1} and **d,** 5000 cd m^{-2}.

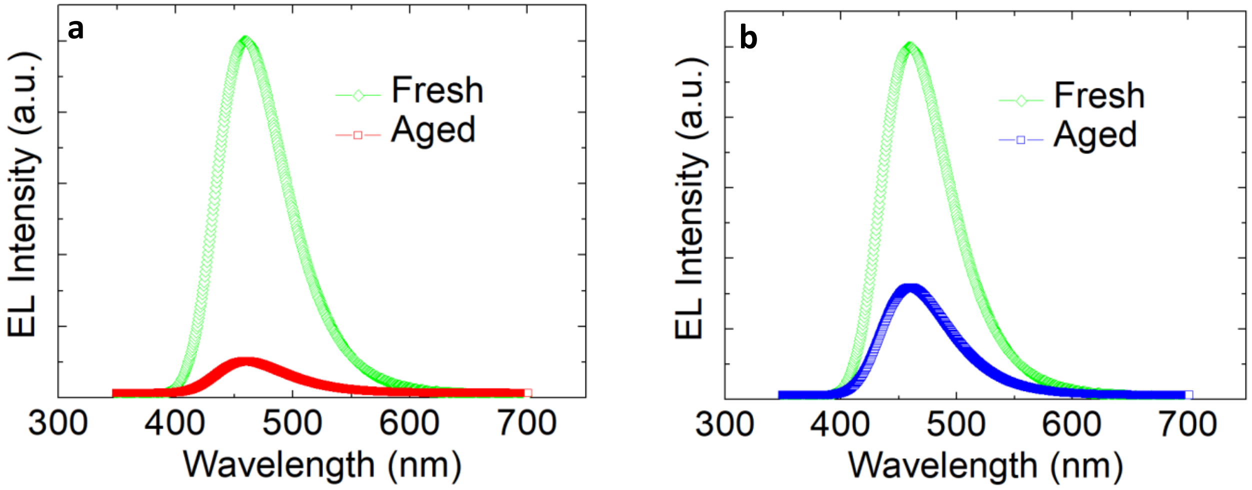

Supplementary Fig. 26 Degradation study. a, Initial and degraded EL spectra of OLEDs using DCDPA as HTL/EBL. **b,** Initial and degraded EL spectra of OLEDs using TQ2f as hole transport/electron blocking material.

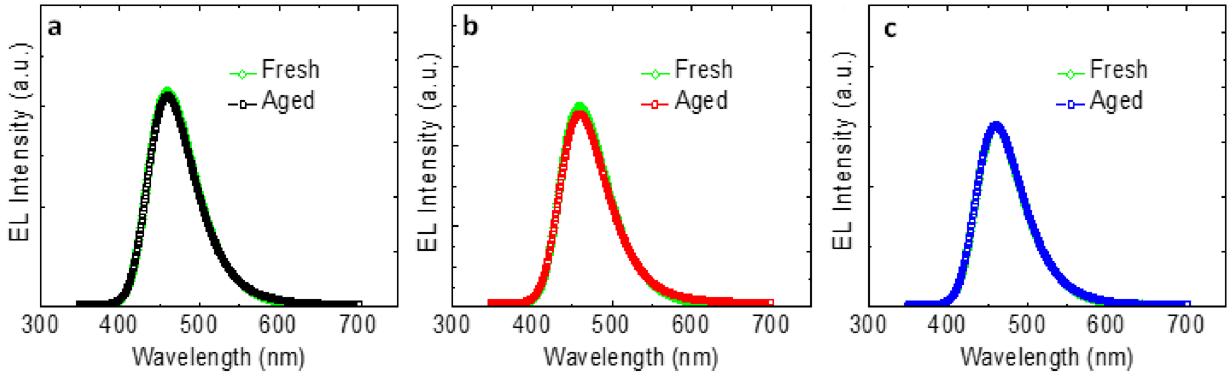

Supplementary Fig. 27 Comparison between fresh and degraded EL spectra. Initial and degraded normalized EL spectra of OLEDs using **a,** T2fQ, **b,** DCDPA and **c,** TQ2f as hole transport/electron blocking material.

Supplementary Table 1 Average molecular weight characteristics of T2fQ and TQ2f

Material	M_n (g/mol)	M_w (g/mol)	PDI
T2fQ	6000	11300	1.9
TQ2f	22600	52500	2.3

Supplementary Table 2 Optical properties of T2fQ and TQ2f

Material	$\lambda_{abs,max}^{sol}$ (nm)	$\lambda_{emm,max}^{sol}$ (nm)	$\lambda_{emm,max}^{film}$ (nm)	HOMO (eV)	LUMO (eV)	E_g (eV)	S_1 (eV)	T_1 (eV)
T2fQ	320 (sh), 538 (max)	600	623	-5.75	-3.71	2.04	2.18	2.14
TQ2f	319, 349 (sh), 524 (sh), 557 (max), 619 (sh)	592	688	-5.70	-3.88	1.82	1.98	1.92

Sol=solution, Sh= shoulder

Supplementary Table 3 Transient PL results. Fitting parameters and exciton lifetime derived from TRPL decay measurements taken on EML films deposited on different substrates

Structure	A_1(%)	τ_1 (ns)	A_2(%)	τ_2 (ns)	$\langle\tau\rangle$ (ns)
Glass/EML	22	1.31	78	4.69	3.94
T2fQ/EML	57	0.85	43	3.41	1.95
DCDPA/EML	66	0.80	34	3.04	1.56
TQ2f/EML	81	0.73	19	2.46	1.06

Supplementary Table 4 Summary of the performance characteristics of OLEDs with the structure ITO/NPD (40 nm)/ T2fQ (15 nm)/TDBA-DI 20% doped in DBFPO (30 nm)/DBFPO (x nm)/TSPO1 (30-x nm)/LiF (1 nm)/Al (100 nm).

X (nm)	λ_{em} (nm)	Turn-on voltage (V)	Maximum luminance (cd m^{-2})	Maximum efficiency (at 1,000 cd m^{-2})[3]		
				CE (cd A^{-1})	PE (lm W^{-1})	EQE (%)
0	458	2.5	49,530	72.19	87.18	41.22
2	458	2.8	40,149	65.43	78.31	37.76
5	458	3.0	37,600	57.10	65.96	33.02
10	458	3.2	25,000	39.23	35.03	23.52

References

1. Singh, R. et al. The impact of thienothiophene isomeric structures on the optoelectronic properties and photovoltaic performance in quinoxaline based donor–acceptor copolymers. *Polym. Chem.* **6**, 3098-3109 (2015).
2. Bronstein, H. et al. Indacenodithiophene-*co*-benzothiadiazole copolymers for high performance solar cells or transistors via alkyl chain optimization. *Macromolecules* **44**, 6649-6652 (2011).
3. Biniek, L. et al. Impact of the alkyl side chains on the optoelectronic properties of a series of photovoltaic low-band-gap copolymers. *J. Polym. Sci. Part A Polym. Chem.* **50**, 1861-1868 (2012).
4. Biniek, L., Fall, S., Chochos, C. L., Leclerc, N., Lévêque, P. & Heiser, T. Optimization of the side-chain density to improve the charge transport and photovoltaic performances of a low band gap copolymer. *Org. Electron.* **13**, 114–120 (2012).
5. Risko, C., McGehee, M. D.& Brédas, J.-L. A quantum-chemical perspective into low optical-gap polymers for highly-efficient organic solar cells. *Chem. Sci.* **2**, 1200-1218 (2011).
6. Chochos, C. L. et al. Experimental and theoretical investigations on the optical and electrochemical properties of *π*-conjugated donor-acceptor-donor (DAD) compounds toward a universal model. *J. Chem. Phys.* **149**, 124902 (2018).
7. G09 | Gaussian.com http://gaussian.com/glossary/g09/ (accessed May 7, 2019).
8. Vecchi, P. A., Padmaperuma, A. B., Qiao, H., Sapochak, L. S., Burrows, P. E. A dibenzofuran-based host material for blue electrophosphorescence. *Org. Lett.* **8**, 4211-4214 (2006).
